# Rice aquaporin OsPIP2;2 is a water‐transporting facilitator in relevance to drought‐tolerant responses

**DOI:** 10.1002/pld3.338

**Published:** 2021-08-16

**Authors:** Jiaqi Bai, Xuan Wang, Xiaohui Yao, Xiaochen Chen, Kai Lu, Yiqun Hu, Zuodong Wang, Yanjie Mu, Liyuan Zhang, Hansong Dong

**Affiliations:** ^1^ College of Plant Protection Shandong Agricultural University Taian China; ^2^ College of Plant Protection Nanjing Agricultural University Nanjing China; ^3^ State Key Laboratory of Crop Biology Shandong Agricultural University Taian China; ^4^ School of Life Sciences Nanjing University Nanjing China; ^5^ Institute of Plant Protection and Agroproduct Safety Anhui Academy of Agricultural Sciences Hefei China

**Keywords:** aquaporin, drought tolerance, PIP2;2, rice, water transport

## Abstract

In rice (*Oryza sativa*), the PLASMA MEMBRANE INTRINSIC PROTEIN (PIP) family of aquaporin has 11 members, OsPIP1;1 to OsPIP1;3, and OsPIP2;1 to OsPIP2;8, which are hypothesized to facilitate the transport of H_2_O and other small compounds across cell membranes. To date, however, only OsPIP1;2, OsPIP2;1, and OsPIP2;4 have been demonstrated for substrate selectivity in their source plant (rice). In this study, OsPIP2;2 was characterized as the most efficient facilitator of H_2_O transport across cell membranes in comparison with the other 10 OsPIPs. In concomitant tests of all *OsPIP*s, four genes (*OsPIP1;3*, *OsPIP2;1*, *OsPIP2;2*, and *OsPIP2;4*) were induced to express in leaves of rice plants following a physiological drought stress, while OsPIP2;2 was expressed to the highest level. After de novo expression in frog oocytes and yeast cells, the four OsPIP proteins were localized to the plasma membranes in trimer and tetramer and displayed the activity to increase the membrane permeability to H_2_O. In comparison, OsPIP2;2 was most supportive to H_2_O import to oocytes and yeast cells. After de novo expression in tobacco protoplasts, OsPIP2;2 exceeded OsPIP1;3, OsPIP2;1, and OsPIP2;4 to support H_2_O transport across the plasma membranes. OsPIP2;2‐mediated H_2_O transport was accompanied by drought‐tolerant responses, including increases in concentrations of proline and polyamines, both of which are physiological markers of drought tolerance. In rice protoplasts, H_2_O transport and drought‐tolerant responses, which included expression of marker genes of drought tolerance pathway, were considerably enhanced by *OsPIP2;2* overexpression but strongly inhibited by the gene silencing. Furthermore, OsPIP2;2 played a role in maintenance of the cell membrane integrity and effectively protected rice cells from electrolyte leakage caused by the physiological drought stress. These results suggest that OsPIP2;2 is a predominant facilitator of H_2_O transport in relevance to drought tolerance in the plant.

## 1 INTRODUCTION

Aquaporins (AQPs) are integral membrane proteins initially defined as “water channels” in all living organisms (Agre, 2004; Agre et al., 1993; Brown, 2017; Preston et al., 1992) but subsequently found to have a broader spectrum of cargo (substrate) selectivity among about 20 compounds (Laloux et al., 2018; Rhee et al., 2017). By the substrate‐transporting role, AQPs participate in many physiological and pathological processes in animals (Brown, 2017; He & Yang, 2019; Bollag et al., 2020; Wang, Schoebel, et al., 2020; Wang, Zhang, et al., 2020) and plants (Laloux et al., 2018; Li et al., 2019; Singh et al., 2020; Tian et al., 2016). For example, if an AQP serves as a H_2_O‐transporting channel, it will be associated with water relations and drought tolerance under most circumstances, possibly in all organs or throughout full life cycle, either in animals (Brown, 2017; de Laurentis et al., 2020; Li & Wang, 2017) or in plants (Hoai et al., 2020; Li & Wang, 2017; Plett et al., 2020; Vishwakarma et al., 2019). In plants, AQPs fall into five major phylogenic families, including the plasma membrane (PM) intrinsic protein (PIP) family. The PIP family is further divided into the PIP1 subfamily, which contains a varied number of PIP1 proteins from PIP1;1 to PIP1;12, and the PIP2 subfamily, which comprises several PIP2 isoforms from PIP2;1 to PIP2;12, in different plant species (Laloux et al., 2018). These proteins are believed to facilitate the transport of different substrates across PMs in an overlapping or redundant manner (Brown, 2017; Maurel et al., 2015a, 2015b). However, substrates transported by only a small number of PIPs have been determined so far, indicating the imminent necessity to characterize substrate selectivity of most PIPs in most plant species, especially food crops (Laloux et al., 2018; Singh et al., 2020).

In food crops, rice (*Oryza sativa*) occupies a prominent position in global food security due to its vast production that is used to feed a huge population in the world. Rice also is representative of cereal crops with respect to the function of PIPs/AQPs in H_2_O transport tightly associated with water utility, osmotic response, and drought tolerance (Afzal et al., 2016; Barzana et al., 2020; Lee et al., 2003; Shekoofa & Sinclair, 2018; Vishwakarma et al., 2019). Drought tolerance is important not only to drylands agriculture (Ayadi et al., 2019; Zhang, Hu, et al., 2019) but also for flooding crops, typically like rice planted in the conventional agriculture (Ding et al., 2015, 2019; Grondin et al., 2016; Henry et al., 2012; Oladosu et al., 2019; Sriskantharajah et al., 2020; Vinnakota et al., 2016). In many countries that possess a large population with relatively little arable land, coastal wetland and salt lick have been reclaimed for rice planting (Chen et al., 2015; Zong et al., 2007; Mañosa et al., 2001; Withers, 2002). Compared with other cereal crops, rice has more excessive transpiration from leaves and lower hydraulic conductance of roots (refer to Nada & Abogadallah, 2020). Therefore, rice is more sensitive to water deficit, to which the water‐transporting role of AQPs/PIPs provides an effective tolerance for survival (Ding et al., 2015, 2019; Grondin et al., 2016; Oladosu et al., 2019; Vinnakota et al., 2016).

Functional multiplicity is a common characteristic of eukaryotic AQPs (Brown, 2017), while PIPs may be more multifaceted in rice than in other plants, especially the biological model *Arabidopsis thaliana*. Rice has almost 36‐fold larger genome size than Arabidopsis but does not have a homolog of *AtPIP1;4* and *AtPIP1;5* (Sakurai et al., 2005). Both rice and Arabidopsis have the same number of members (PIP2;1 to PIP2;8) in the PIP2 subfamily, but the number of members is different in the PIP1 subfamily. The PIP2 subfamily is consisting of AtPIP1;1 to AtPIP1;5 in Arabidopsis but only has OsPIP1;1 to OsPIP1;3 in rice (Laloux et al., 2018). Presumably, OsPIPs have a higher degree of functional multiplicity. To date, however, OsPIPs have received poor attentions in contrast to extensive understandings of the homologs in other plants with respect to the fundamental importance for substrate transport (Bezerra‐Neto et al., 2019; Kromdijk et al., 2020) and functional regulation mechanisms (Kukulski et al., 2005; Kirscht et al., 2016; Wang, Wang, et al., 2018; Singh et al., 2020). While several PIPs have been characterized as transport channels for specific substrates in biological model plants like Arabidopsis and cereal crops like barley (Fox et al., 2017; Sadura et al., 2020), only three OsPIPs were studied with respect to substrate selectivity in their source plant (rice) (Table 1). OsPIP1;2 was recently shown to support mesophyll CO_2_ transport and phloem sucrose transport (Xu et al., 2019). This protein was identified as a physiologically relevant facilitator of CO_2_ transport across PMs of rice cells based on gas exchange measurements performed on leaves of the wild‐type (WT) and *OsPIP1;2*‐overexpressing rice plants (Xu et al., 2019). However, direct evidence is still lacking to demonstrate the role of OsPIP1;2 in mediating sucrose transport through the phloem PMs or sieve plates. OsPIP1;4 was characterized as an H_2_O‐transporting channel by analyzing root hydraulic conductivity and xylem sap flow in the WT and *OsPIP2;4*‐overexpressing rice plants (Nada & Abogadallah, 2020). OsPIP2;1 was demonstrated to be a H_2_O‐transporting facilitator based on measurements of hydraulic conductivity in root cells of the WT and *OsPIP2;1*‐silenced rice plants (Ding et al., 2019). In contrast, substrate selectivity of OsPIP1;1, 1;3, 2;2, 2;3, 2;5, 2;6, and 2;8 was tested in yeast but not in their source plant (Table 1).

**TABLE 1:** Rice PIPs characterized for substrate selectivity and physiological or pathological relevance

OsPIPs	Substrates	Study systems	Evaluation criteria	Regulated processes	References
1;1, 1;2, 1;3 2;1, 2;2, 2;4, 2;6, 2;8	None tested	Rice	Gene expression levels	Drought tolerance assumed	Grondin et al., 2016
2;1, 2;1, 2;2, 2;3, 2;4, 2;5	H_2_O	Yeast	Stopped‐flow spectrometry	Osmotic response	Sakurai et al., 2005, 2008
1;3, 2;3, 2;4, 2;5	None tested	Rice	Gene expression levels	Chilling response	Sakurai et al., 2005, 2008
2;1	CO_2_	Rice	Curve‐fitting	Photosynthesis	Xu et al., 2019
1;2	H_2_O	Yeast	Membrane water permeability	Water relations	Ding et al., 2019
		Rice	Root hydraulic conductivity	Water relations	
2;4	H_2_O	Rice	Root hydraulic conductivity and xylem sap flow	Water relations	Nada & Abogadallah, 2020

Abbreviation: PIP, plasma membrane intrinsic protein.

We have extensively investigated plant PIPs with a main attempt to elucidate their implications in plant growth‐defense tradeoffs (Zhang, Chen, & Dong, 2019). Emerging evidence demonstrates that several PIPs have the dual functions in both physiological and pathological processes, not only regulating plant growth and development (Li et al., 2015, 2019) but also partaking in plant infection by and immunity against pathogens (Bian et al., 2019; Ji & Dong, 2015; Li et al., 2019; Tian et al., 2016; Wang, Schoebel, et al., 2020; Wang, Zhang, et al., 2020; Zhang, Chen, & Dong, 2019; Zhang, Hu, et al., 2019). We compared OsPIPs in terms of physiological performances in response to the bacterial blight pathogen *Xanthomonas oryzae* pv. *oryzae* (*Xoo*) when it was infecting plants of the susceptible rice variety Nipponbare (Ji & Dong, 2015a; Wang, Zhang, et al., 2018; Li et al., 2019). We found that OsPIP1;4 can be used by *Xoo* to translocate a virulent effector from the bacteria into rice cells, wherein the bacterial effector activates a disease‐susceptible gene to induce virulence in Nipponbare plants (Wang, Zhang, et al., 2018; Bian et al., 2019; Li et al., 2019; Zhang, Hu, et al., 2019). This finding confirms the recently proposed conceptional revolution that functions of AQPs far exceed the originally defined category only for substrate transportation (Hara‐Chikuma et al., 2015; Afzal et al., 2016; Ha, 2017; Li et al., 2019). However, it was unclear whether the OsPIPs that associate with plant infection and immunity have a primary role for substrate transport in rice plants growing under regular conditions without pathogen infection.

In the present study, we analyzed OsPIPs with respect to their presence or absence in H_2_O transport across living cell PMs according to the original definition of “water channels” (Agre, 2004; Agre et al., 1993; Brown, 2017; Preston et al., 1992). We further determined the role of OsPIP2;2 in the plant resistance to a physiological drought stress caused by Polyethylene glycol 6,000 (PEG_6000_). PEG with molecular mass 4,000–8,000 effectively induces physiological drought in a variety of plant species, and PEG_6000_ has been mostly used (Arisha et al., 2020; Dong et al., 2005; Hajihashemi & Geuns, 2016; Huang et al., 2019; Ren et al., 2019; Tiwari et al., 2020; Zhang et al., 2017). While PEG induces drought syndromes from leaf wilting to plant collapse, plants in this process may defend themselves by activating the drought tolerance pathway to preserve water relations (Dong et al., 2005; Zhang et al., 2017). By cytological, physiological, and molecular assays, we show that OsPIP2;2 is a predominant facilitator of H_2_O transportation in relevance to drought‐tolerant responses in rice cells.

## 2 MATERIALS AND METHODS

### 2.1 Plant growth and treatment

Seeds of the rice variety Nipponbare were initially provided by Professor Hongsheng Zhang (Nanjing Agricultural University) and then reproduced and maintained in this lab. Seeds of tobacco *(Nicotiana benthamiana*) were reproduced and maintained in this lab. Rice and tobacco seeds were germinated in flat plastic trays filled with a substrate containing an industrial soil branded as PINDSTRUP. Five days later, the germinal seedlings were moved into 12‐L pots (two plants per pot) filled with the same soil. Seeds were incubated, and the plants were grown in environment‐controlled chambers under 28°C, 12‐h light at 250 ± 50 μmol quanta/m^2^/s and a relative humidity of 80%.

PEG_6000_ was used as an inducer of physiological drought stress (Dong et al., 2005; Dubois & Inzé, 2020) and applied to 15‐day‐old rice seedlings. Usually, PEG_6000_ is prepared as an aqueous solution at a certain percent concentration (w/v) and applied in parallel to the deionized water control to incubate plants by immersing the root system. In this study, 15‐day‐old rice seedlings growing in the PINDSTRUP soil were taken out carefully by pushing the soil away using a finger, cleaned gently by soft washing with tap water to remove the surrounding soil scraps, and then placed into plastic tubes containing deionized water. After a 12‐h acclimation, seedlings were moved into new tubes containing deionized water (control) or an aqueous solution of PEG_6000_ justified to 0%, 5%, 10%, 15%, 20%, 25%, and 30% w/v, respectively. Seedlings were monitored in the subsequent 10 h by automatic photography at 10‐min intervals. Chronological development of drought syndromes was analyzed.

### 2.2 Quantification of *OsPIP* expression levels

Total RNA was isolated from leaves and subjected to real‐time quantitative reverse polymerase chain reaction (qRT‐PCR). All the qRT‐PCR experiments were performed with the QuantStudio®3 Real‐Time PCR Instrument (ThermoFisher Scientific) and using the constitutively expressed *EF1α* gene as a reference (Liu et al., 2011). Relative expression level of a tested gene was quantified as the tested gene to *EF1α* transcript quantity ratio, which was determined by the 2^−ΔΔ*C*^_T_ method (Livak & Schmittgen, 2011).

### 2.3 *OsPIP* de novo expression

#### 2.3.1 Expression in oocytes

**FIGURE 1:**
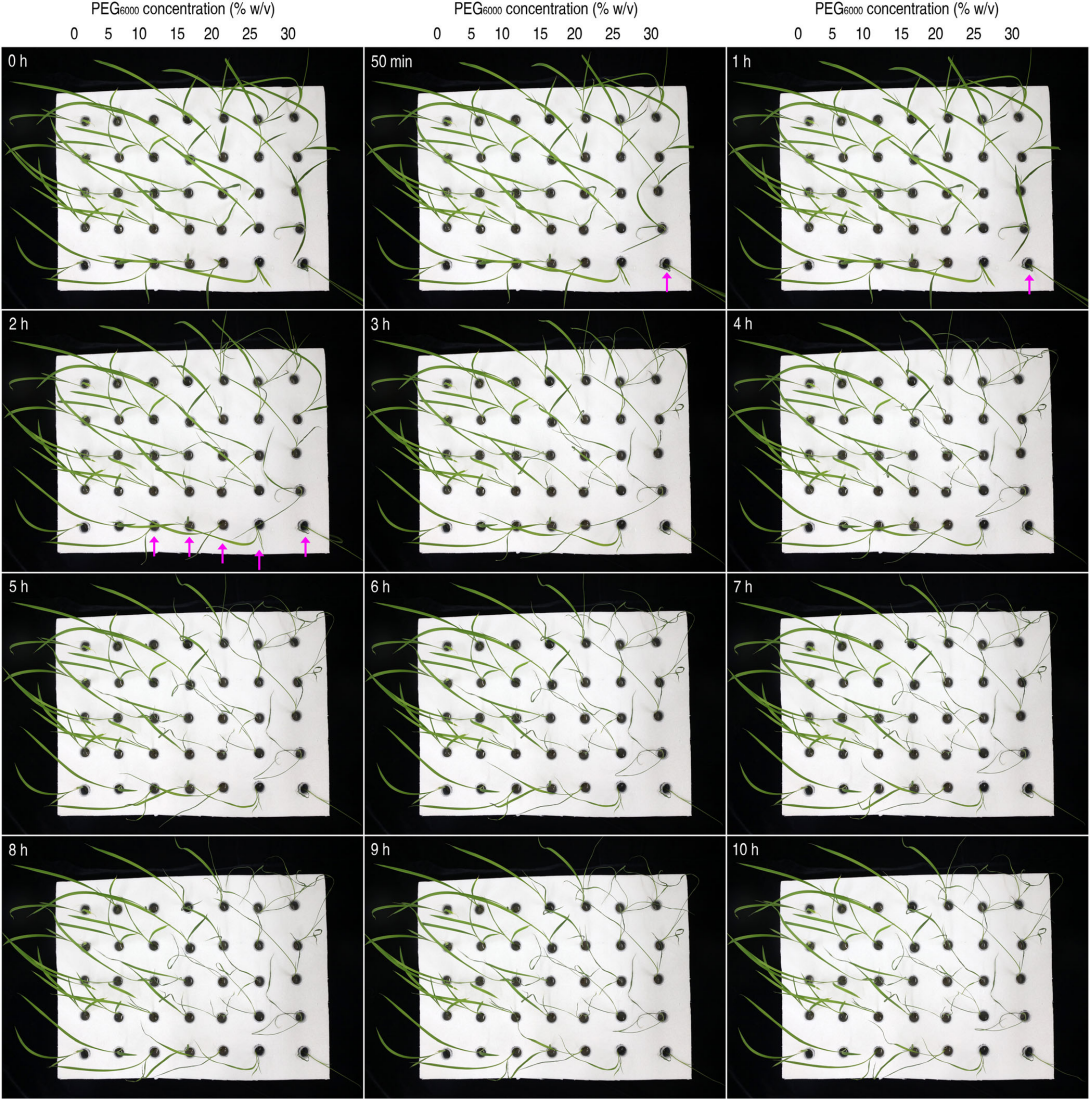
Chronological changes in rice response to physiological drought stress caused by PEG_6000_ applied in a range of concentration. Fifteen‐day‐old rice seedlings were incubated by root immersion in deionized water containing PEG_6000_ added at the indicated concentrations. Images were obtained by automatic photography in 10 h at 10‐min intervals and are partially shown here on the hour except one image in which seedlings incubated with the highest concentration of PEG_6000_ had displayed drought syndromes. Arrowheads point the concentrations that already induced drought syndromes. Each image represents 105 plants treated proportionally with each of the 7 PEG_6000_ doses and tested in 3 independent experiments

Based on gene expression analyses (Figure 1), *OsPIP1;3*, *OsPIP2;2*, *OsPIP2;2*, and *OsPIP2;4* were further investigated. Complementary DNAs (cDNAs) of *OsPIPs* were obtained by RT‐PCR amplification of total RNAs isolated from leaves of 15‐day‐old rice plants. For use in transformation of African clawed frog (*Xenopus laevis*) oocytes, every OsPIP cDNA was fused to the *enhanced green‐fluorescent protein* (*eGFP*) gene or a six‐*his* tandem sequence by conventional recombination methods (Li et al., 2015, 2019). Commercial nursery *X*. *laevis* was obtained from Stem Cell Bank of Chinese Academy of Sciences and maintained in an 18°C water incubator supplied twice a week with blood worm and ox heart, liver, and lung. Mature oocytes at V to VI stages were excised and used in transformation. Briefly, the binary vector pGH19 and an *eGFP*‐fused *OsPIP* cDNA were digested with *Xba*I and *Eco*R1 (TAKARA) and then glued with T4 ligase (Thermo Fisher). The recombinant vector was linearized with restriction enzyme *Not*I (TAKARA) and purified in RNase‐free water. A constant amount of 1 μg linearized DNA was applied for an in vitro transcription using RiboMAX™ Large‐Scale RNA Production Systems‐T7 (Promega). The resulting cRNA was injected into oocytes. The transformed oocytes were incubated in sterile ND96 solution (96‐mM NaCl, 2.0 mM KCl, 1.8 mM CaCl_2_, 1.0 mM MgCl_2_·6H_2_O, 5.0 mM HEPES, 2.0‐mM Na‐pyrnvate, pH 7.6) amended with penicillin and streptomycin. After incubation for 36 h, oocytes were observed between 495 and 520 nm using 488‐nm argon‐ion laser excitation with a Zeiss LSM700 laser scanning confocal microscope. Fusion proteins were analyzed by immunoblotting of membrane proteins isolated from the transformed oocytes using a previously described protocol (Jørgensen et al., 2016). Similar procedure was used in construction, de novo expression, and analyses of the OsPIP‐his fusion protein except for laser scanning confocal microscopy (LSCM).

#### 2.3.2 Expression in yeast

Each of the *OsPIP‐his* fusion genes was cloned into the yeast binary vector pYES2 (Wang, Zhang, et al., 2020) with the help of restriction enzymes *Hin*dIII and *Eco*RI (TAKARA). The recombinant vector was transformed into competent cells of the wine‐brewing yeast (*Saccharomyces cerevisiae*) strain NMY51 in transformation solution (.1 M LiCl, 30% w/v PEG_4000_). The positive transformants were cultured in SD‐Ura (synthetic dextrose minimal media without uracil) media with 2% w/v galactose overnight at 30°C and harvested in phosphate buffer solution (.2 mM, pH 7.4). Membrane proteins were isolated from the transformed yeast cultures using a previously described protocol (Hansen et al., 2017) and analyzed by immunoblotting. Yeast cells were observed between 495 and 520 nm using 488‐nm argon‐ion laser excitation with a Zeiss LSM700 laser scanning confocal microscope.

#### 2.3.3 Expression in tobacco plants

De novo expression of the selected *OsPIP*s (*OsPIP1;3*, *OsPIP2;1*, *OsPIP2;2*, and *OsPIP2;4*) in leaves of tobacco was performed by the agroinoculation method (Liu et al., 2011). Each of the *OsPIP* genes was combined at the N‐terminus with the constitutive 35S promoter from cauliflower mosaic virus and at the C‐terminus with the *YELLOW‐FLUORESCENT PROTEIN* (*YFP*) gene from a previously used yeast vector (Li et al., 2015). The fusion gene was inserted into the plant binary vector pCAMBIA1301 (Liu et al., 2011). The recombinant vector was transferred into competent cells of the *Agrobacterium tumefaciens* strain GV3101. A bacterial suspension of the recombinant GV3101 cultures was infiltrated into intercellular spaces of fully expanded leaves of 30‐day‐old tobacco plants. In the subsequent 48–60 h, transinfected leaves were excised and observed by LSCM to reveal the subcellular localization of OsPIP‐YFP fusion proteins.

#### 2.3.4 Expression in plant protoplasts

This was performed by the chemical mediation method (Shen et al., 2014). Protoplasts were isolated from strips of fully expanded leaves of 30‐day‐old tobacco seedlings or from stems and leaf sheaths of 30‐day‐old rice plants, using reagents from Sigma‐Aldrich and a previously described protocol (Shen et al., 2014; Yoo et al., 2007). Cell walls were removed by fungal cellulase and macerozyme used in an aqueous solution. The enzyme solution containing released protoplasts was diluted with equal volume of aqueous washing solution (154‐mM NaCl, 125‐mM CaCl_2_ and 5‐mM KCl, 2‐mM 4‐morpholineethanesulfonic acid, and pH 5.7) and filtered with a 75‐μm nylon mesh. The filtrate collection was centrifuged at 100*g* and 4°C for 2 min. The supernatant was discharged. The precipitate was resuspended with the washing solution and centrifuged at 1000*g* and 4°C for 5 min. The supernatant was removed again. Tubes containing the protoplasts were placed on ice bath and suspended with the MMg solution (4‐mM 2,4‐morpholino‐ethanesulfonic acid, .4‐M mannitol, and 15‐mM MgCl_2_) and adjusted to 5 × 10^6^ protoplasts/ml. This protoplast suspension was supplied with 10 μl of the recombinant vector that carries an *OsPIP* insert, 110 μl of PEG‐Ca^2+^ (40% w/v PEG4000, .2‐M mannitol, and 1‐M CaCl_2_), followed by incubation on ice bath for 30 min. At this time point, the transformation was terminated by adding 440 μl of the W5 solution (4‐mM MES, .5‐M mannitol, and 5‐mM KCl). The suspension of transformed protoplasts was centrifuged at 1000*g* and 4°C for 5 min, the supernatant was removed, and the WI solution (4‐mM MES, .5‐M Mannitol, and 5‐mM KCl) was gently added. The resulting protoplast suspension was shifted into wells of a 96‐well plate, incubated for 12 h under room temperature and weak illumination, and used in further tests.

### 2.4 *OsPIP2;2* silencing and overexpression

The rice tungro bacilliform virus (RTBV) vector pRBDV (Kant & Dasgupta, 2017; Purkayastha et al., 2010) was used in hairpin and overexpression constructions (Li et al., 2019). The hairpin unit was constructed using the gene internal 300‐bp fragment from the less conserved 241–553 region of the *OsPIP2;2* coding sequence (Figure S1). The specificity of the *OsPIP2;2*_241–553_ fragment in targeting the endogenous gene was verified by hybridization predominantly with *OsPIP2;2* over the other 10 *OsPIP*s (Figure S2). The *OsPIP2;2*_241–553_ sequence was cloned by RT‐PCR using RNA isolated from leaves of rice plants 3 h after incubation in 30% w/v PEG_6000_ and then inserted into pRBDV. This yielded the recombinant vector carrying the pRBDV:*OsPIP2;2*_241–553_ sense and pRBDV:*OsPIP2;2*_241–553_ antisense sequences, respectively. Overexpression of *OsPIP2;2* was constructed by inserting the 35S promoter and *OsPIP2;2* CDS fusion gene into pRBDV, generating the recombinant pRBDV:*OsPIP2;2* vector. Subsequently, each recombinant vector was transferred into the tobacco genome by transfecting leaves of 20‐day‐old tobacco plants. Fifteen days later, the gene silencing and overexpression efficiencies were confirmed by RT‐PCR analyses using RNA isolated from leaves newly growing in the transfected plants. Leaf transformation with the hairpin construct resulted in 40%–80% fall in the *OsPIP2;2* transcript quantity in experimental replicates. The *OsPIP2;2*‐silenced plants were used in protoplast preparation and water permeability assays only when the gene silencing efficiency reached to 70% or higher. In experimental replicates, leaf infection with the overexpression construct caused 55%–87% enhancement in *OsPIP2;2* expression. The *OsPIP2;2*‐overexpressing plants were used in protoplast preparation and water permeability assays only when the gene overexpression efficiency reached to 75% or higher.

### 2.5 Physiological measurements

#### 2.5.1 Water permeability measurements

Water permeability of the directly transformed rice and tobacco protoplasts, protoplasts from the transformed oocytes and yeast, and protoplasts from the *OsPIP2;2*‐silenced and ‐overexpressing rice plants was measured by microscopy. These protoplasts were separately incubated in the ND96 solution, which provides a low osmotic gradient from exteriors to interiors of the incubated protoplasts. Ten minutes later, the osmotic water permeability coefficient (Pf) was determined by measuring volume changes of the protoplasts as previously described (Ding et al., 2019; Li et al., 2015).

#### 2.5.2 Proline measurement

Proline concentrations in tobacco and rice protoplasts were determined with a plate reader. Before testing, a standard curve was established using a commercial proline standard of known concentrations and the corresponding absorbance readings. To isolate proline from protoplasts, 5 ml of 3% sulfosalicylic acid solution was added into a tube containing a protoplast suspension and then the sample was setting for 10 min in a boiling water bath. The extraction solution was filtered into a clean test tube after cooling and the supernatant was regarded as a proline extract. This proline extract solution was mixed with equal volumes of glacial acetic acid and acid ninhydrin. After the mixture was phased automatically, solution of the upper phase was collected and centrifuged at 3,500*g* for 5 min. Supernatant was assayed for absorbance at 520 nm in the plate reader. Proline concentration in the supernatant was estimated with reference to gradients of the absorbance by methylbenzene. The proline content was given as micrograms per gram protoplasts.

#### 2.5.3 Polyamine measurement

A previously described protocol (Zhu et al., 2020) was used to extract polyamines (PAs) from rice and tobacco protoplasts, respectively. Soluble conjugated PAs were calculated by subtracting the free PAs from the acid‐soluble PAs. Precipitated protoplasts were suspended with 4 ml of v/v 5% cold perchloric acid (PCA) and incubated at 4°C for 1 h, followed by sonication in the presence of lyase and antifoam. The resulting suspension was centrifuged at 12,000*g* and 4°C for 10 min. The supernatant was supplied with the internal standard 1,6‐hexanediamine, and the mixture was centrifuge at 12,000 *g* and 4°C for 30 min. The resulting supernatant was blended with 6‐N HCl at a 1:5 volume rate and hydrolyzed at 110°C for 18 h in flame‐sealed glass ampules. After acid hydrolysis, HCl was evaporated by heating at 70°C, and the residue was suspended in 2 ml of 5% PCA, followed by centrifugation at 12,000*g* and 4°C for 30 min. The supernatant contained the acid‐soluble PA fraction and free Pas liberated from PA conjugates. To obtain the insoluble bound PA, the pellets were rinsed four times with 5% PCA to remove any traces of soluble PAs and were suspended in 5 ml of 6 N HCl. This solution was hydrolyzed by the same procedure as described above. PAs recovered from the nonhydrolyzed supernatant, hydrolyzed supernatant, and the pellet were benzoylated as follows. An aliquot of the supernatant was treated with 2 ml of 2 N NaOH and 15 ml of benzoyl chloride, vortexed vigorously, and incubated for 30 min at 37°C. The reaction was terminated by adding 4‐ml saturated NaCl solution. Thereafter, the benzoyl PAs were extracted with 3‐ml cold diethyl ether. Finally, 1.5 ml of the ether phase was evaporated to dryness and redissolved in 1‐ml methanol. PAs in the final solution were assayed by high‐performance liquid chromatography (HPLC) (Agilent 1220, America). Ten microliters of benzoyl PAs in methanol solution was injected into a 4.6 × 250‐mm reverse‐phase Kromasil C18 column (Agilent, Sweden) at 25°C. PAs were eluted from the column with 64% methanol at a flow rate of .7 ml/minute. PA peaks were detected with the plate reader at 254 nm.

#### 2.5.4 Electrolyte leakage measurement

Electrolyte leakage from leaves of rice seedlings was measured using a conductivity meter. Briefly, 1‐cm leaf segments were immersed into deionized water by the aid of a vacuum pump, followed by incubation for 1 h under 25°C. The resulting solution containing electrolytes leaked from leaf segments were subjected to measurements with the Leitz DDS‐307 conductivity meter (Shanghai Right‐One Instruments Company, LTD) under 220 V, 50 Hz, and 1.021 Kohlrausch coefficient. Similar measurements on deionized water were conducted in control. Extents of electrolyte leakage from WT, *OsPIP2;2*‐Si, and *OsPIP2;2*‐OE were given as the measured conductivity (μS/cm) values.

### 2.6 Data treatment

All experiments were repeated at least three times with similar results. Quantitative data were analyzed using the commercial IBM SPSS19.0 software package (Shi, 2012). Homogeneity‐of‐variance in data was determined by the Levene test. The formal distribution pattern of the data was confirmed by the Kolmogorov–Smirnov test and P–P Plots. Fisher's data‐pairing test or Duncan's new multiple range test was performed along with analysis of variance (ANOVA) on data from at least three independent experiments, each involving three repetitions, unless specified elsewhere, such as when a leaf was treated as a statistical unit.

## 3 RESULTS

### 3.1 *OsPIP2;2* highly responds to a physiological drought stress

**FIGURE 2:**
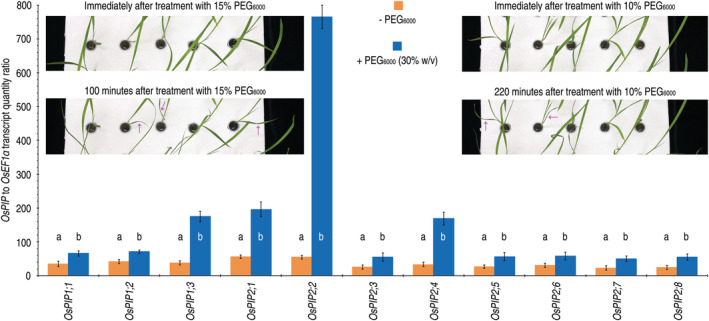
Expression levels of *OsPIP*s in rice plants temporally incubated in deionized water with and without supply with PEG_6000_. Rice seedlings already growing for 15 days in pot soil in a plant growth chamber were shifted into tubes containing deionized water. After 12‐h acclimation, these plants were transferred into new tubes containing deionized water only or an aqueous solution of PEG_6000_ at the indicated concentration. Insets show morphological changes of seedlings growing under the indicated conditions. Gene expression was analyzed by QRT‐PCR performed with total RNAs isolated from the aerial parts of the plants 3 h after incubation. The constitutively expressed *OsEF1α* gene was used as a reference to quantify relative expression level of an *OsPIP*. Data show are mean values ± standard deviation (SD) estimates. The number of experimental repeats (*n*) = 6 independent experiments each involving 15 plants tested in three biological repeats. Different letters on graphs indicate significant differences by analysis of variance (ANOVA) and Fisher's test between the pairs of data (*P* = .001–1.2 × 10^−7^)

To identify OsPIP candidates implicated in water relations of rice, we analyzed expression levels of 11 *OsPIP* genes in plants of the rice variety Nipponbare following PEG_6000_ treatment (Figure 1). We applied a range of PEG_6000_ concentrations, 0%–30% at 5% gradients, to 15‐day‐old rice seedlings in liquid culture, and monitored chronological development of drought syndromes in the subsequent 10 h (Figure 1; Table 2). In that period, the lowest PEG_6000_ dosage (5%) caused no responses, but drought syndromes were induced when PEG_6000_ concentration was increased to 10% and higher (Figure 1). Leaf lengthening and narrowing (Figure 2, insets) were found to be the first sign of drought syndromes, occurred mostly on the second leaves (Figure 2, insets) in 50–110 min after PEG6000 application at 10%–30% (Table 2). The formation of slender leaves was thought to indicate plant transition from the normal physiological status to drought response. This transition was followed by wilt of partial to all leaves and plant collapse in the end (Figures 1 and 2). All these syndromes occurred in 6 h after plant incubation with 25% and 30% PEG_6000_ supplies, respectively (Table 2), but concentrations of 15% and 10% only caused partial leaves to wilt.

**TABLE 2:** Chronological changes in response of 15‐day‐old rice seedlings to a range of PEG_6000_ dosage in liquid culture

Drought syndrome development timing (min)		PEG_6000_ concentration (% w/v)					
	0	05	10	15	20	25	30
Time to first occurrence of leaf lengthening	NO	NO	110	100	90	60	50
Time to first occurrence of leaf wilt	NO	NO	250	230	120	100	70
Time to wilting of all leaves	NO	NO	NO	NO	220	180	150
Time to plant collapse	NO	NO	NO	NO	NO	320	280

Abbreviation: NO, not occur.

To evaluate the effects of the physiological stress on *OsPIP* expression, an aqueous solution of 15% or 30% PEG_6000_ was applied to 15‐day‐old rice seedlings by immersing roots. These plants (+ PEG_6000_) and those incubated in deionized water (−PEG_6000_) were used 3 days later in *OsPIP* expression assessments by qRT‐PCR. The qRT‐PCR analyses using total RNA isolated from the leaves indicated that all *OsPIP*s responded to the physiological drought stress, displaying significantly (*P* = 1.2 × 10^7^) enhanced expression in contrast to the steady‐state expression levels in control (Figure 2, graph). Extents by which the physiological drought stress‐induced gene expression were 3.6, 2.5, and 4.1 times for *OsPIP1;3*, *OsPIP2;1*, and *OsPIP2;4*, respectively, reached 12.9‐fold for *OsPIP2;2*, but the extents were much smaller (1.7–88.7% times) for additional OsPIPs. Evidently, *OsPIP2;2* exceeds all the other *OsPIP* genes in response to the physiological drought stress and displays the highest level of induced expression under the stress condition, indicating that OsPIP2;2 is likely to take part in water relations of rice.

### 3.2 De novo expression of *OsPIP2;2* enhances water permeability of oocyte and yeast membranes

**FIGURE 3:**
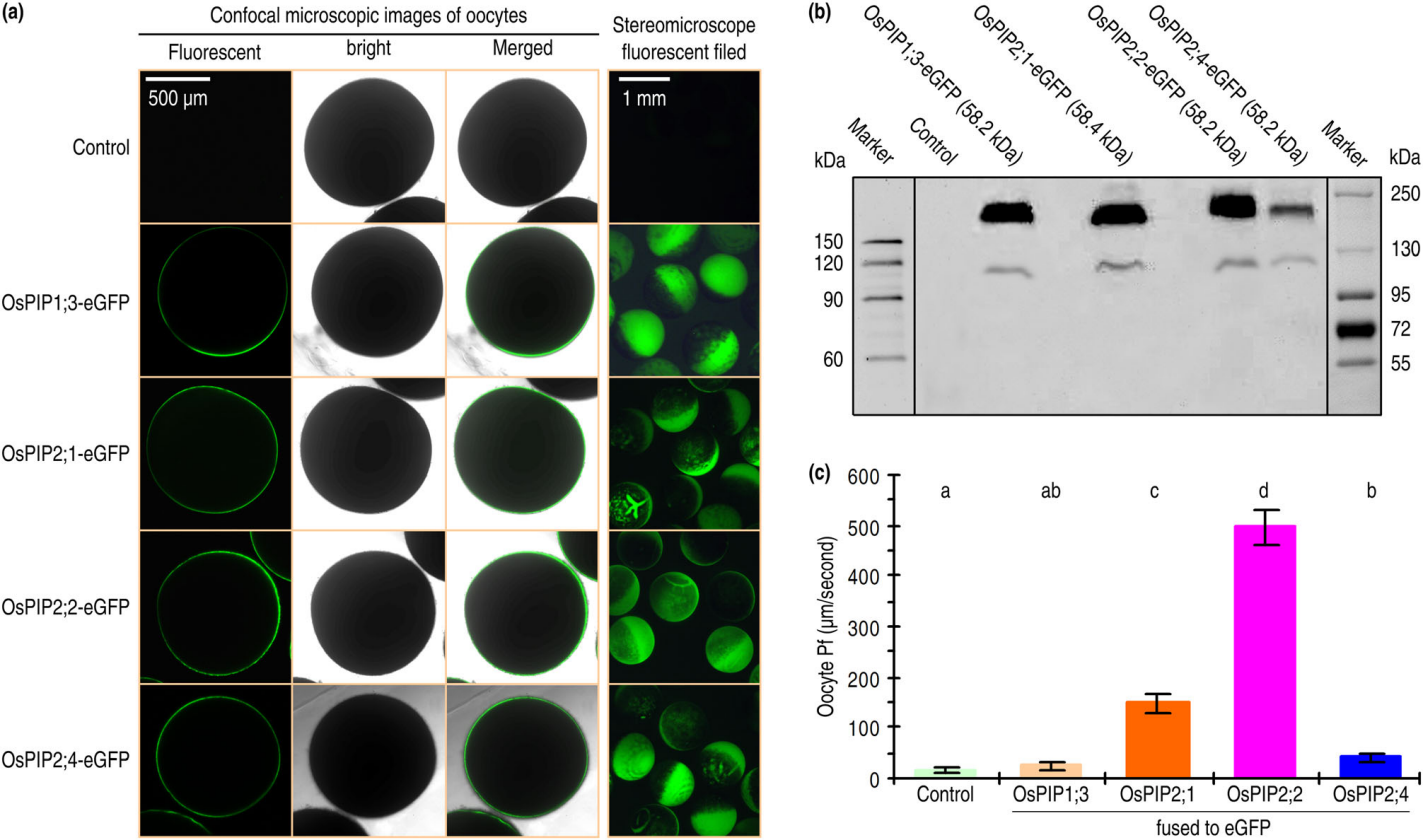
De novo expression of *OsPIP‐eGFP*s in African clawed frog oocytes and the subsequent effects on osmotic water permeability (Pf) of the oocyte membranes. (a) Microscopic observations on oocytes 48 h after transformation with the recombinant vector containing *OsPIP‐eGFP* fusion genes or with the empty vector (control) that did contain any gene insert. Each image represents 60 oocytes transformed in 6 independent experiments. (b) Immunoblotting of oocyte proteins hybridized with the GFP antibody. (c) Pf measurements. Data shown are mean values ± SDs of results from 6 independent experiments each involving 10 oocytes (*n* = 60). Different letters on graphs indicate significant differences based on ANOVA and Duncan's multiple new multiple range test of the data obtained from the different oocytes (*P* =.056–1.5 × 10^−16^)

Based on the conceptual function of AQPs initially defined for water transport (Agre, 2004), we tried to determine if *OsPIP1;3*, *OsPIP2;1*, *OsPIP2;2*, and *OsPIP2;4* have a role in mediating H_2_O transport in living cells of both the wine‐brewing yeast and African clawed frog oocytes. LSCM performed on oocytes 48 h after transformation indicated that all the fusion proteins (OsPIP1;3‐eGFP, OsPIP2;1‐eGFP, OsPIP2;2‐eGFP, and OsPIP2;4‐eGFP) were localized to oocyte membranes, in contrast to the absence of any protein fluorescence in control (Figure 3a). Every OsPIP‐eGFP fusion protein was plentiful in the transformed oocytes, as determined by stereomicroscopic observations (Figure 3a) and immunoblotting analyses (Figure 3b), respectively. These proteins were detected mainly as tetramer, accompanied by trimer at a small quantity (Figure 3b).

**FIGURE 4:**
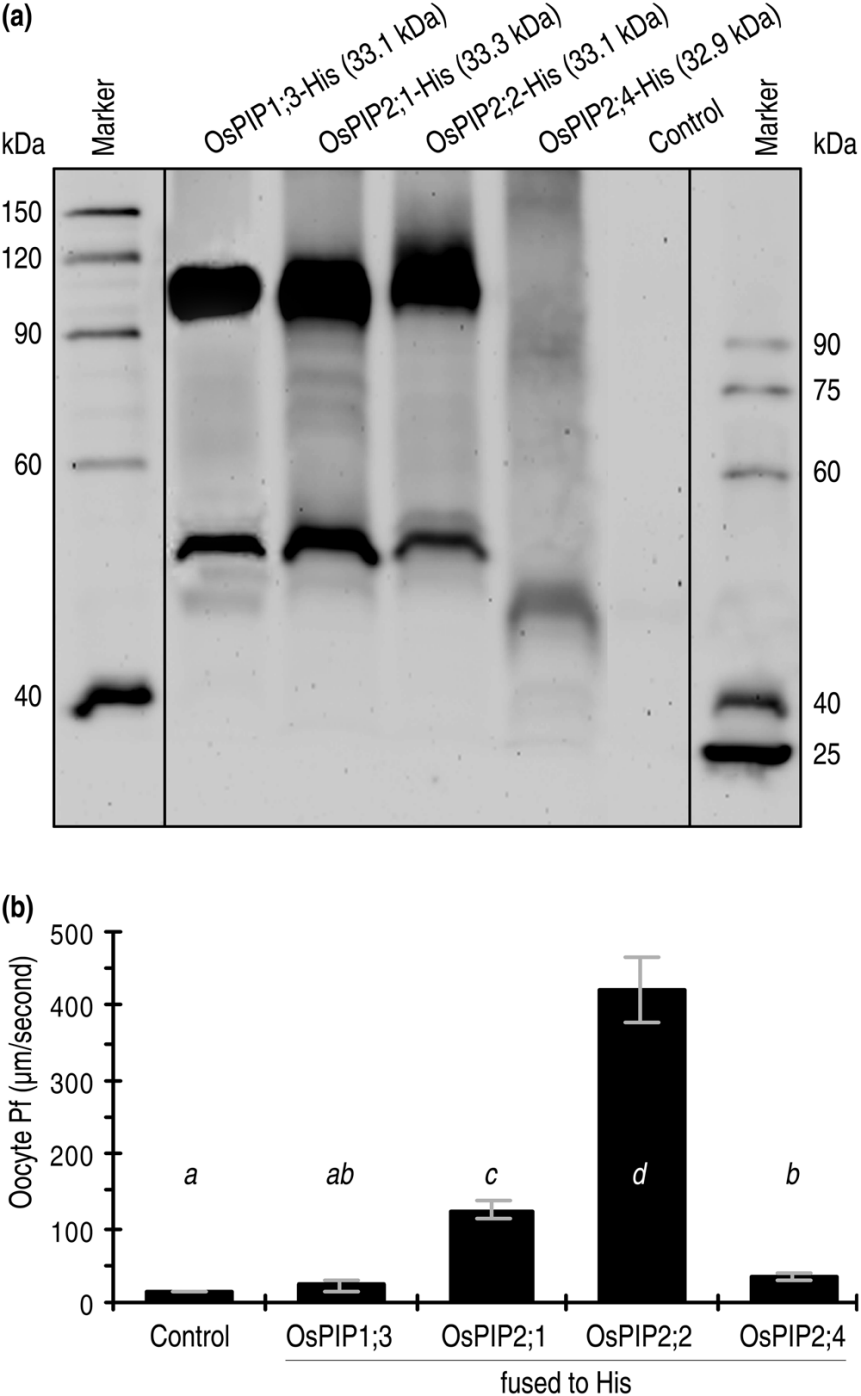
De novo expression of OsPIP‐His fusion proteins in African clawed frog oocytes and the subsequent effects on Pf of the oocyte membranes. (a) Immunoblotting of oocyte proteins hybridized with the His antibody. (b) Pf measurements. Data shown are mean values ± SDs of results from 6 independent experiments each involving 10 oocytes (*n* = 60). Different letters on graphs indicate significant differences based on ANOVA and Duncan's multiple new multiple range test of the data obtained from the different oocytes (*P* = .056–1.2 × 10^−17^)

**FIGURE 5:**
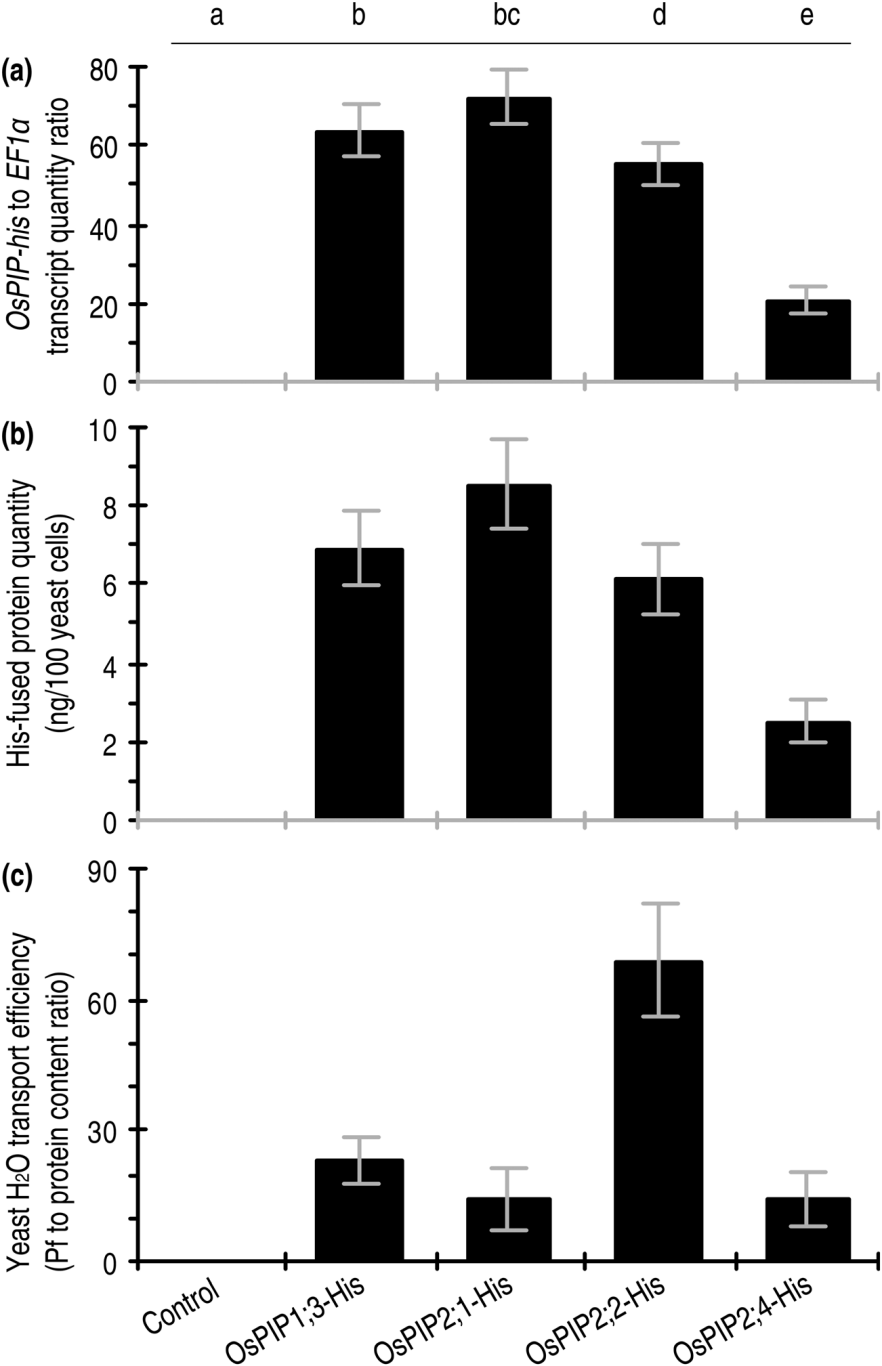
De novo expression of *OsPIP‐His* fusion genes in yeast and the subsequent effects on Pf of the yeast membranes. (a) Relative levels of *OsPIP* expression in yeast cultures 24 h after transformation with the recombinant vector containing an insert of the *OsPIP*‐his fusion genes in comparison with the empty vector (control). (b) Quantification of OsPIP‐His fusion proteins based on immunoblotting of yeast proteins hybridized with the His antibody. (c) Pf measurements. In a to c, data shown are mean values ± SDs of results from 6 independent experiments. Different letters on graphs indicate significant differences based on ANOVA and Duncan's multiple new multiple range test of the data (*P* = .038–1.2 × 10^−17^)

Based on the measured Pf values measured under a low extracellular osmotic gradient, OsPIP2;2‐eGFP greatly promoted transport of the environmental H_2_O into cells of the oocytes transformed with *OsPIP2;2‐eGFP* in contrast to eGFP (Figure 3c). OsPIP2;1‐eGFP was weaker in this function, while neither OsPIP1;3‐eGFP nor OsPIP2;4‐eGFP exhibited evident effect on the water permeability of oocyte membranes (Figure 3c). In line with this result, plentiful production of the OsPIP‐His fusion proteins was detected from the OsPIP1;3‐his–transformed oocytes (Figure 4a). Once again, all the OsPIP‐His fusion proteins existed in both tetramer and trimer (Figure 4a). While OsPIP2;2‐His and OsPIP2;1‐His caused a high and a moderate elevation in Pf, respectively, neither OsPIP1;3‐His nor OsPIP2;4‐His caused evident changes in Pf values over the basal level found in control (Figure 4b). Then, the *OsPIP‐His* genes were expressed (Figure 5a) and the fusion proteins produced (Figure 5b) in yeast protoplasts. When the recombinant yeast protoplasts were incubated under a low osmotic gradient, Pf was increased by each OsPIP‐His compared to His only, but the increase level was greater in yeast protoplasts that produced OsPIP2;2‐His than those that produced the other OsPIP‐His proteins (Figure 5c). These results suggest that de novo expression of *OsPIP2;2* in oocyte and yeast cells facilitates H_2_O transport across the cell PMs.

### 3.3 *OsPIP2;2* de novo expression promotes H_2_O transport across tobacco PMs

**FIGURE 6:**
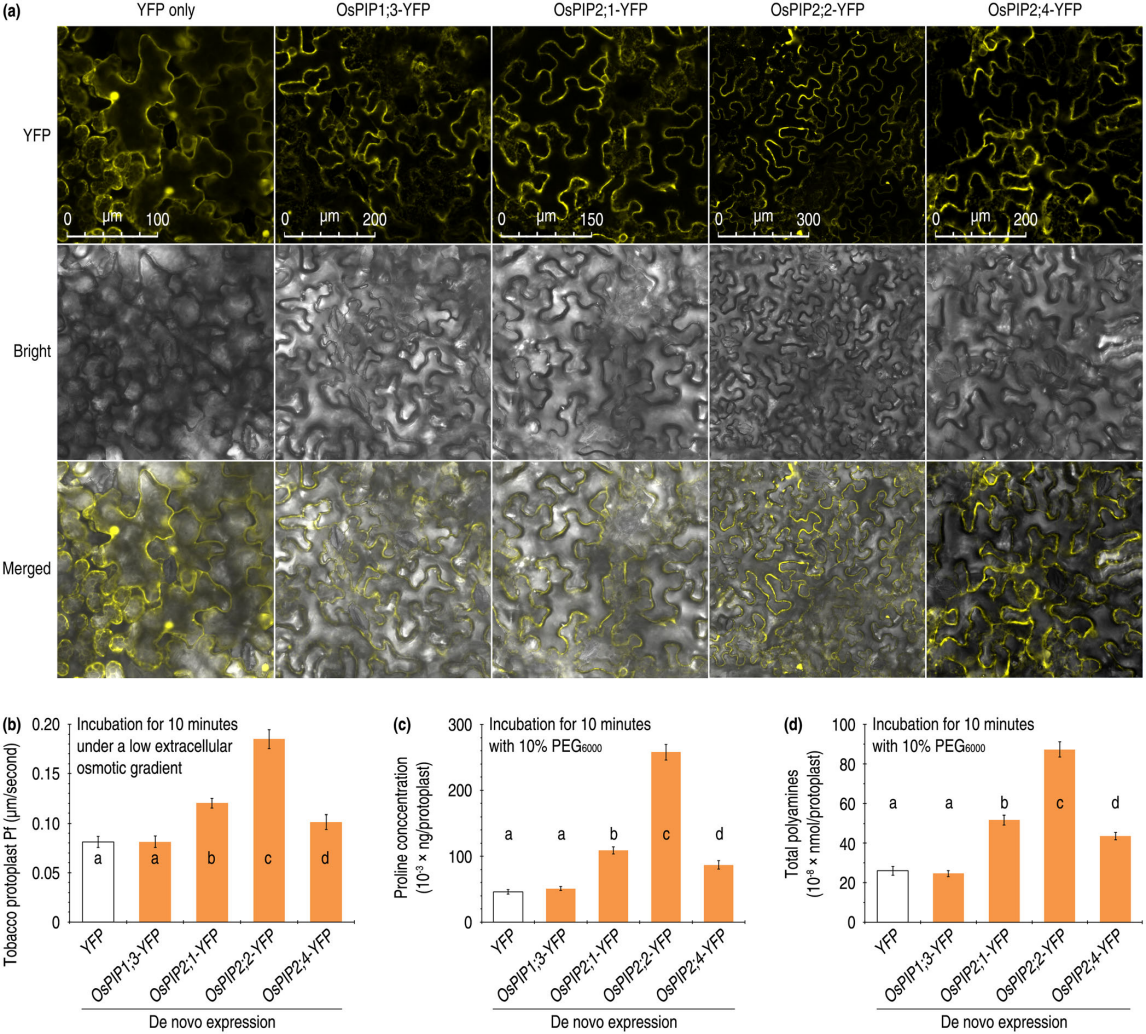
De novo expression of OsPIP‐YFP with effects on Pf and drought‐tolerant responses in tobacco. (a) Subcellular localization of YFP and OsPIP‐YFP fusion proteins visualized by laser scanning confocal microscopy (LSCM) on leaves of tobacco plants 48 h after transformation with the corresponding genes. Each image represents 18 leaves from nine plants. (b) Pf measurements under the annotated conditions. (c, d) Concentrations of proline and polyamines in protoplasts incubated under the indicated conditions. In b to d, data shown are mean values ± SDs of results from six independent experiments. Different letters on graphs indicate significant differences based on ANOVA and Duncan's multiple new multiple range test of the data (*P* = 1.235–1.6 × 10^−9^)

**FIGURE 7:**
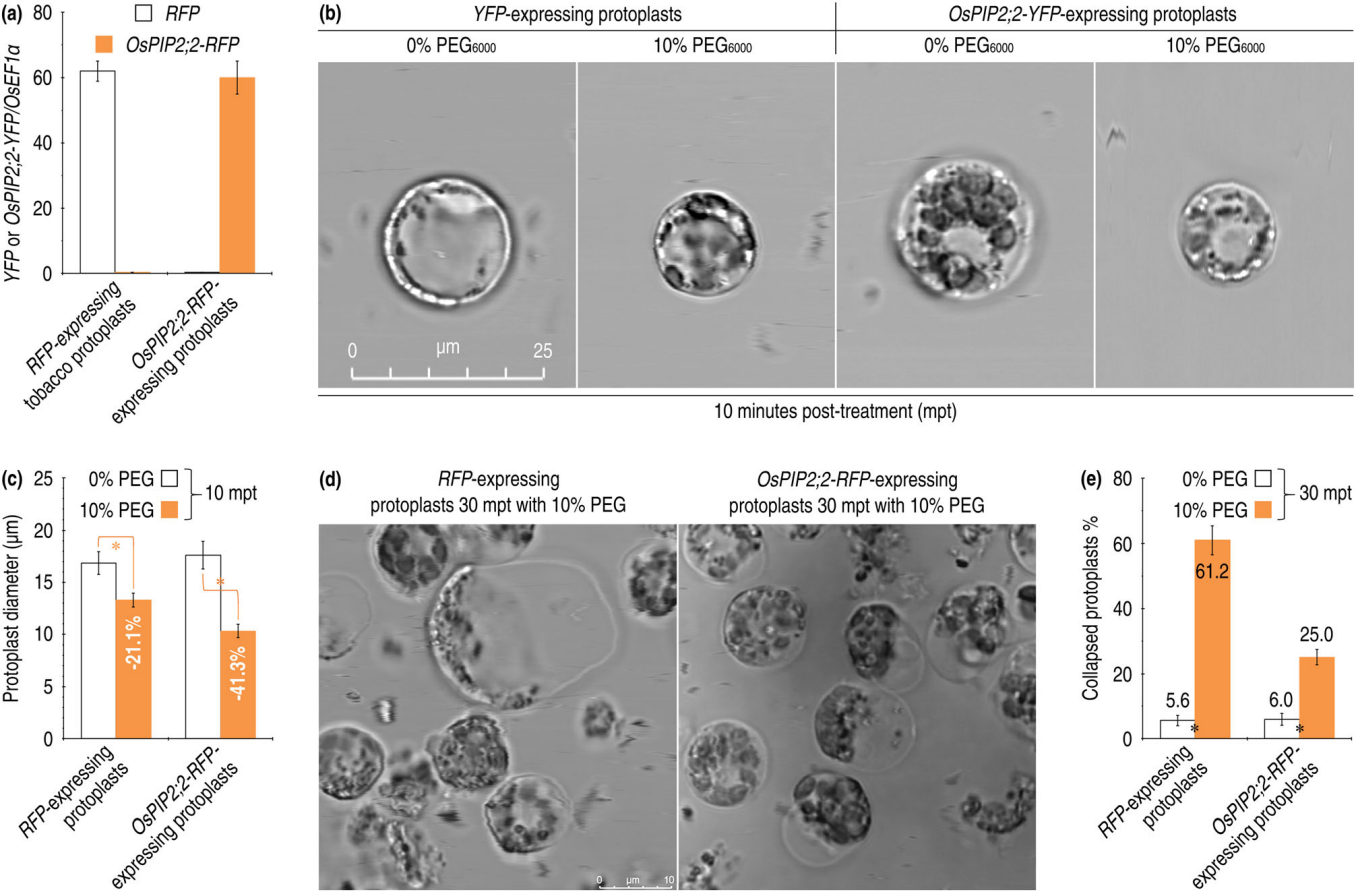
Morphological changes of tobacco protoplasts in response to PEG_6000_ treatment. (a) Microscopic images of the protoplasts. (b) Quantification of protoplast diameters. (c) Microscopic images showing collapse of partial protoplasts. (d) Quantification of malformed protoplasts. In b and d, each image represents about 3,000 protoplasts observed in 3 independent experiments. In c and e, data shown are mean values ± SDs of results from 3 independent experiments. Asterisks indicate significant differences between the pairs of data based on ANOVA and Fisher's test (*P* = .005–1.6 × 10^−9^)

We turned to study cytological and physiological performances of OsPIP1;3, OsPIP2;1, OsPIP2;2, and OsPIP2;4 in plants. LSCM performed on tobacco leaves 48 h after transformation with the *OsPIP‐YFP* genes clearly detected the production of OsPIP1;3‐YFP, OsPIP2;1‐YFP, OsPIP2;2‐YFP, and OsPIP2;4‐YFP fusion proteins in the leaf cells (Figure 6a). The four OsPIP‐YFP fusion proteins were all localized to PMs of the leaf cells, in contrast to apparently triple distributions of YFP only in the PMs, cytoplasmic spaces, and nuclei (Figure 6a). The protein production was correlated with expression of the *YFP* and OsPIP1;2‐YFP genes (Figure 7a).

To assess if OsPIP1;3, OsPIP2;1, OsPIP2;2, and OsPIP2;4 affect H_2_O transport through plant PMs, tobacco protoplasts were transformed with the recombinant pCAMBIA1301 vector containing *YFP* (control) and *OsPIP‐YFP*, respectively. When transformed protoplasts were incubated in the ND98 medium that held an extracellularly low osmatic gradient, Pf increases over the control level were substantially induced by OsPIP2;1‐YFP, OsPIP2;2‐YFP, and OsPIP2;4‐YFP (Figure 6b). In comparison, OsPIP2;2‐YFP displayed the most vigorous activity, providing the highest level in Pf elevation. On the contrary, OsPIP1;3‐YFP did not cause evident effect on Pf (Figure 6b). Thus, OsPIP2;2 is a vigorous performer in mediating H_2_O transport across PMs of tobacco cells under the de novo expression condition.

### 3.4 *OsPIP2;2* de novo expression contributes to drought‐tolerant responses in tobacco protoplasts

We analyzed if any of OsPIP2;1‐YFP, OsPIP2;2‐YFP, and OsPIP2;4‐YFP affects drought‐tolerant responses in tobacco protoplasts incubated with PEG_6000_. Increases in concentrations of proline and total PAs (including putrescine, spermidine, and spermine) are regarded as a physiological indicator of drought tolerance activation in plants (Oladosu et al., 2019; Vinnakota et al., 2016; Zhu et al., 2020). Thus, the transformed protoplasts were incubated with 10% PEG_6000_, and proline and PA concentrations were measured 10 min later. At this time point, protoplast concentrations of both proline (Figure 6c) and PAs (Figure 6d) were significantly (*P* = 1.6 × 10^−9^) increased by OsPIP2;1‐YFP, OsPIP2;2‐YFP, and OsPIP2;4‐YFP in contrast YFP only. In these fusion proteins, OsPIP2;4‐YFP displayed the greatest activity in increasing proline and PAs, but OsPIP1;3‐YFP had little effect on concentrations of both compounds (Figure 6c,d).

In addition to the physiological responses, cytological variations also occur in plants under a drought stress (Dong et al., 2004; Oladosu et al., 2019). We confirmed that the PEG_6000_ treatment (tobacco protoplast incubation in the presence of 10% PEG_6000_) caused considerable reductions in protoplast size, indicating protoplast contraction due to water outflux (Figure 7b,c). Protoplast contraction became evident in 10 min posttreatment (mpt) with PEG_6000_, as compared to PEG‐absent control (Figure 7b). Compared to control, the PEG treatment induced significant (*P* = 1.6 × 10^−9^) decreases in diameters of the protoplasts no matter if they had been transformed with *YFP* only or with the *OsPIP2;2‐YFP* fusion gene (Figure 7c). However, de novo expression of *OsPIP2;2‐YFP* (Figure 7a) aggravated protoplast contraction to a higher level, reducing protoplast diameter by a 20% higher degree than that of *YFP* only (Figure 7c). Intriguingly, de novo expression of *OsPIP2;2‐YFP* seemed to intensify the integrity of protoplast membranes (Figure 7d,e). Protoplasts started to swell after 10 mpt, when the cytoplasm was congregated at one side, and partially collapsed not until 30 mpt (Figure 7d). At 30 mpt, the *YFP*‐expressing protoplasts were malformed at a 61.2% proportion on average, whereas average collapse proportion remained as low as 25.9% in the protoplasts expressing *OsPIP2;2‐YFP* (Figure 7e). These analyses suggest that de novo expression of *OsPIP2;2* enhances drought‐tolerant responses in tobacco cells.

### 3.5 *OsPIP2;2* promotes H_2_O transport and drought‐tolerant responses in rice protoplasts

**FIGURE 8:**
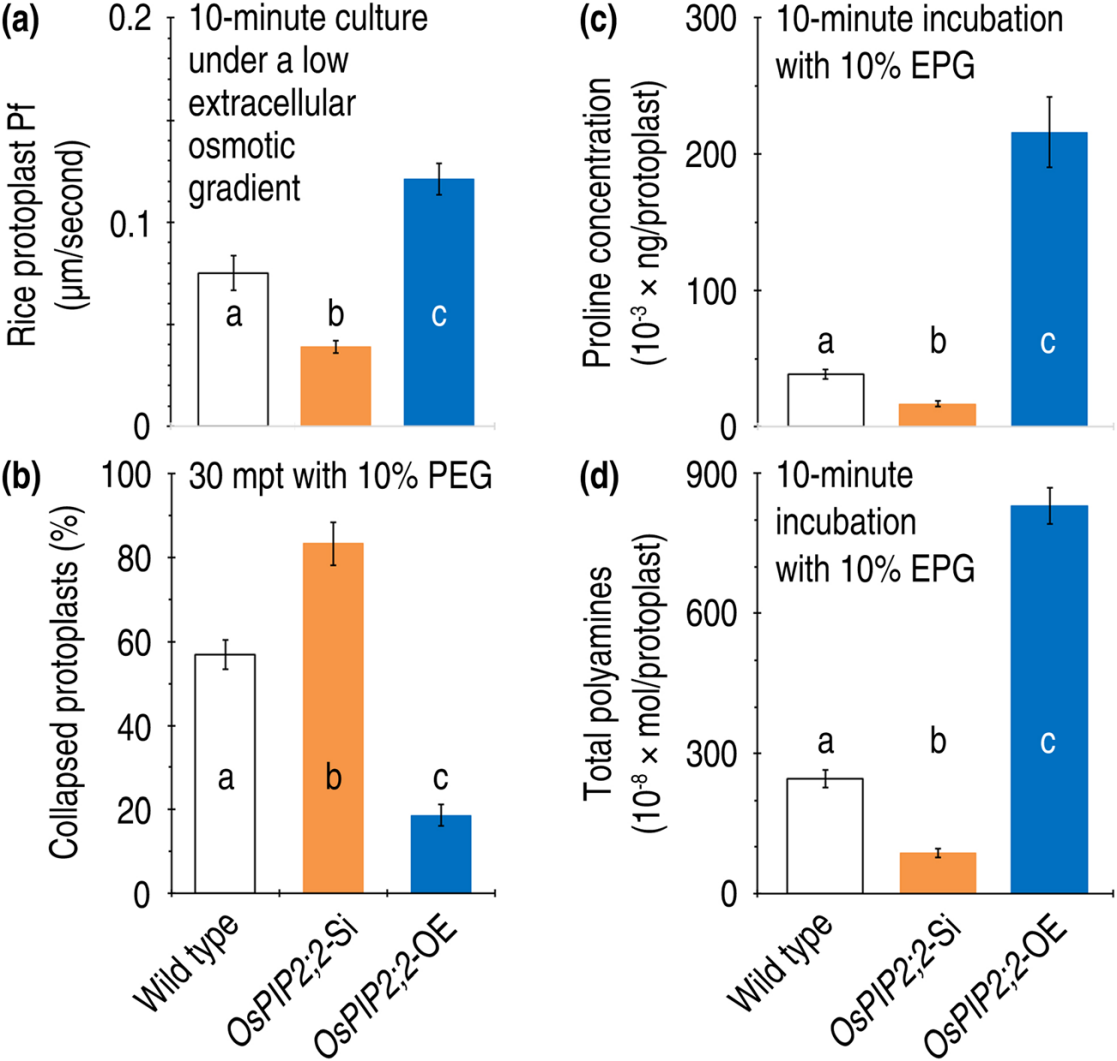
The effects of *OsPIP2;2* silencing and overexpression on physical and physiological responses to PEG_6000_ treatment in rice protoplasts. (a) The protoplast Pf measurements. (b) Quantification of the protoplast decomposition. (c) and (d) Concentrations of proline and polyamines in the protoplasts. In a to d, data shown are mean values ± SDs of results from six independent experiments. Different letters on graphs indicate significant differences based on ANOVA and Duncan's multiple new multiple range test of the data (*P* = 1.8 × 10^−7^–1.2 × 10^−16^)

We analyzed H_2_O transport and drought‐tolerant responses in rice protoplasts prepared from the Nipponbare plants that express the canonical *OsPIP2;2* gene (WT) or display *OsPIP2;2* silencing (*OsPIP2;2*‐Si) or overexpression (*OsPIP2;2*‐OE) mediated by the RBDV vector (Kant & Dasgupta, 2017; Li et al., 2019). By measuring Pf in protoplasts incubated in the liquid medium with a low osmotic gradient, we verified that considerable amounts of H_2_O moved into the protoplast interiors over 10 min. In that period, quantities of H_2_O transport across rice protoplast PMs were significantly (*P* = 1.2 × 10^−16^) increased by *OsPIP2;2*‐OE but decreased by *OsPIP2;2*‐Si compared to the canonical expression (Figure 8a). After 10% PEG_6000_ was supplied to the medium, rice protoplasts became malformed in 30 min. In comparison to the WT, the *OsPIP2;2*‐Si protoplasts collapsed at a higher proportion, but the collapse rate was substantially reduced by *OsPIP2;2*‐OE (Figure 8b). Consistently, drought‐tolerant responses, indicated by protoplast concentrations of proline (Figure 8c) and PA (Figure 8d), were induced at a greater level in the *OsPIP2;2*‐OE protoplasts compared to the WT. On the contrary, *OsPIP2;2*‐Si caused significant reductions in concentrations of proline (Figure 8c) and PA (Figure 8d). Clearly, OsPIP2;2 functions in protoplasts of its source plant to facilitate H_2_O transport across the PMs and contributes to defense responses against the physiological drought stress.

### 3.6 *OsPIP2;2* conduces to membrane integrity of rice protoplasts

**FIGURE 9:**
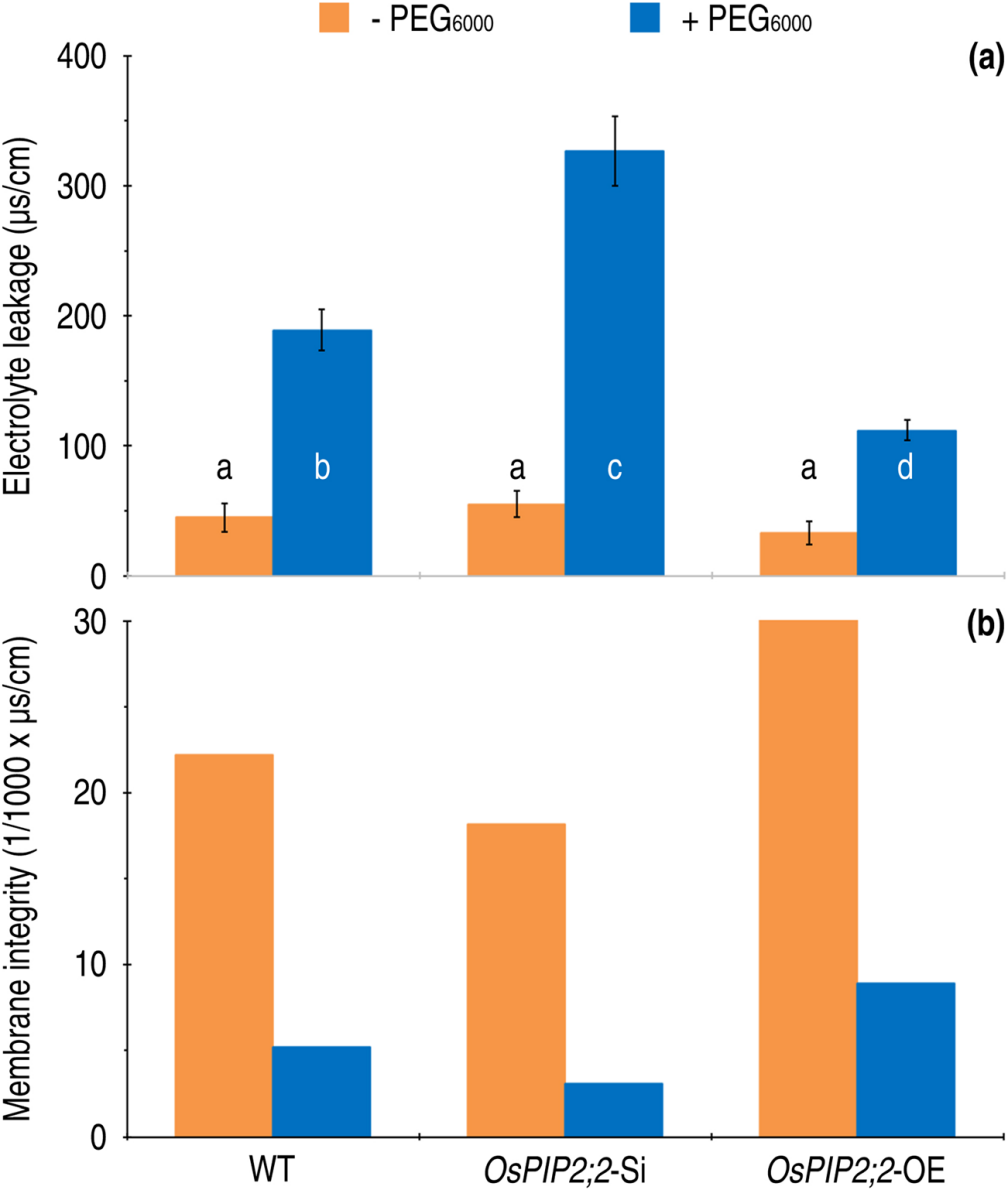
The effects of *OsPIP2;2* silencing and overexpression on cell membrane integrity of 15‐day‐old rice seedlings growing in the absence (−) and presence (+) of PEG_6000_ treatment. (a) Electrolyte leakage from leaf segments of the plants 3 h after PEG_6000_ treatment or remained free from PEG_6000_. Data shown are mean values ± SDs of results from six independent experiments. Different letters on graphs indicate significant differences based on ANOVA and Duncan's multiple new multiple range test of the data (*P* = .875–1.5 × 10^−17^). (b) Rice cell membrane integrity given as reciprocals of electrolyte leakage calculated as averages based on data from a

In plants, one of PEG‐induced responses is cellular ion efflux known as electrolyte leakage, which occurs due to injured integrity of the PMs (Dong et al., 2005). We analyzed whether the role of *OsPIP2;2* in reducing rice protoplast collapse (Figure 8b) relates to its effect on electrolyte leakage from the protoplasts in response to PEG_6000_. When the WT, *OsPIP2;2*‐Si, and *OsPIP2;2*‐OE plants incubated in deionized water were supplied with 30% PEG_6000_ and measured 3 h later, electrolyte leakage from leaves was detected at different extents. Compared to the WT, the *OsPIP2;2*‐OE plants had much less electrolyte leakage, but electrolyte leakage from *OsPIP2;2*‐Si plants was significantly increased (Figure 9a). Conveying the conductivity values to thousand times of their reciprocals reflects integrity degrees of the protoplast membranes (Figure 9b), suggesting that *OsPIP2;2* is conducive to maintenance of the membrane integrity in response to the physiological drought stress.

### 3.7 *OsPIP2;2* imparts activation of the drought tolerance pathway

**FIGURE 10:**
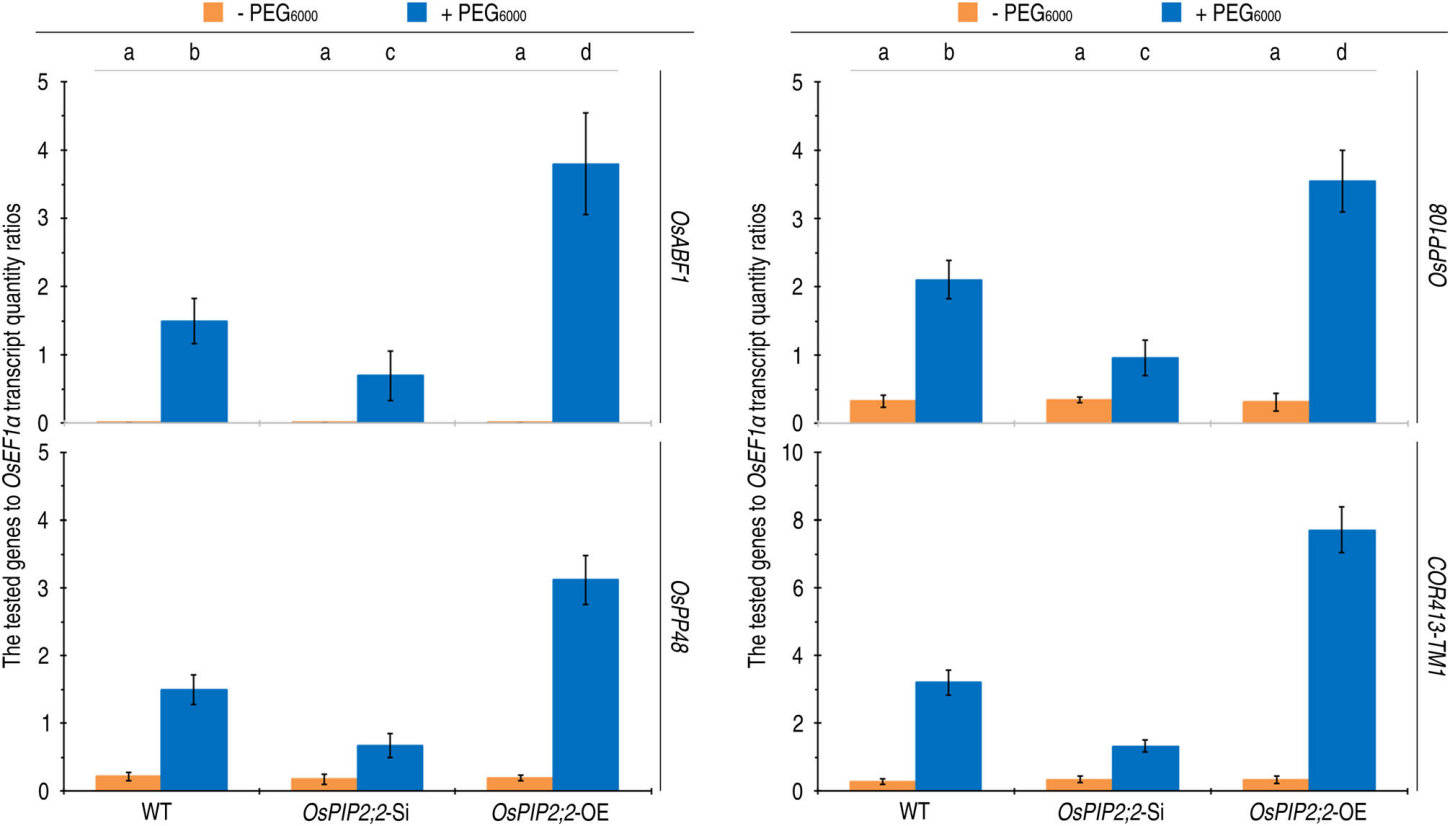
The effects of *OsPIP2;2* silencing and overexpression on expression of response genes regarded as molecular makers of the drought tolerance pathway. Rice seedlings already growing for 15 days in pot soil in a plant growth chamber were shifted into tubes containing deionized water. After 12‐h acclimation, these plants were transferred into new tubes containing deionized water only or an aqueous solution of 30% PEG_6000_. Three hours later, gene expression was analyzed by QRT‐PCR performed with total RNAs isolated from the aerial parts of the plants. The constitutively expressed *OsEF1α* gene was used as a reference to quantify relative expression level of an *OsPIP*. Data show are mean values ± SD estimates. Different letters on graphs indicate significant differences by analysis of variance (ANOVA) and Duncan's multiple new multiple range test of the data (*P* = .0001–1.7 × 10^−11^; *n* = 6 independent experiments each involving 15 plants tested in three biological repeats)

In rice, activation of the drought tolerance pathway by PEG treatment essentially involves induced expression of the pathway‐marker gene *COR413‐TM1* and the pathway‐regulatory genes *OsABF1*, *OsPP48*, and *OsPP108* (Zhang et al., 2017). We determined that these genes were induced by PEG_6000_ treatment to markedly express in leaves of the WT, *OsPIP2;2*‐Si, and *OsPIP2;2*‐OE plants (Figure 10). As quantified at 3 h after PEG_6000_ application, levels of *COR413‐TM1*, *OsABF1*, *OsPP48*, and *OsPP108* expression were significantly (*P* < .001) induced in contrast to the steady‐state expression levels found in the PEG‐absent control plants. Moreover, *COR413‐TM1* exceled *OsABF1*, *OsPP48*, and *OsPP108* in expression extents in all plants. Noticeably, the *OsPIP2;2*‐OE plants highly exceeded but *OsPIP2;2*‐Si was considerably inferior to the WT in supporting PEG_6000_‐induced expression of all the genes (Figure 10). Clearly, *OsPIP2;2* imparts the drought tolerance pathway toward activation in response to the physiological drought stress.

## 4 DISCUSSION

Rice has a total of 33 AQPs including 11 PIP homologs (Sakurai et al., 2005) that mostly have not been characterized regarding substrate selectivity and specificities, as well as associated bioprocesses (Table 1). By quantifying differential expression of all the *OsPIP* homologs in rice plants responding to the physiological drought stress induced by the externally applied PFG_6000_, we elucidated that *OsPIP1;3*, *OsPIP2;1*, *OsPIP2;2*, and *OsPIP2;4* are potentially involved in water relations and drought tolerance in the plant. Based on measurements of membrane permeability to H_2_O in the *OsPIP*‐transformed oocyte and yeast cells, we showed that OsPIP2;2 is a predominant facilitator for H_2_O transport across living cell membranes. By physiological and cytological analyses of plant protoplasts, we demonstrated that OsPIP2;2 is an effective implementor of H_2_O transport across PMs in its source plant (rice). This is a small but substantial advance in exploring substrate selectivity and specificities of rice PIPs. The paucity in understandings of rice PIPs is incredible if we do not have the opportunity to scrutinize previous literatures. Previously to the present study, only three OsPIPs have characterized clearly to execute the substrate‐transporting function in their source plant (Table 1).

Previously, OsPIP2;1 was identified as a physiologically relevant CO_2_‐transporting implementor in rice (Xu et al., 2019). In rice, moreover, both OsPIP1;2 (Ding et al., 2019) and OsPIP2;4 (Nada & Abogadallah, 2020) were characterized to support root hydraulic conductivity. It is unclear whether OsPIP1;2 and OsPIP2;4 also contribute to leaf hydraulic conductivity, which can be detected by cell pressure probing measurements (Hachez et al., 2011) as reliable as for the root (Li et al., 2015). No evidence has been obtained to elucidate whether an OsPIP functions for substrate transport in both roots and leaves, but it is frequent that a particular AQP plays a same role in different organs of plants (Gomes et al., 2009; Li et al., 2015; Maurel et al., 2008). Our study identifies OsPIP2;2 to be the third recognized but most vigorous H_2_O‐transporting facilitator among 11 PIP homologs of rice. In yeast and oocytes, OsPIP2;2 and OsPIP2;4 contribute relatively smaller parts to H_2_O transport in contrast to the prominent role of OsPIP2;2. This result is consistent with the regular pattern of functional redundancy when two or more AQP homologs fulfill a same function but functional intensities are different one from another (Gomes et al., 2009; Maurel et al., 2008; Péret, et al., 2012). Our results also agree with previous studies by Sakurai and colleagues (Sakurai et al., 2005, 2008). They did not test substrate‐importing functions of any OsPIPs in rice. Instead, they analyzed differential expression of 33 AQP‐encoding genes including 11 *OsPIP*s, which constitute the full repertoire of AQPs in the rice genome, in different organs of rice plants after chilling treatment (Sakurai et al., 2005). Then, they performed de novo expression assays in a yeast (*S*. *cerevisiae* strain BJ5458) system and characterized OsPIP2;2 as a strong moderator for H_2_O import into cells of the *PIP*‐transformed yeast cultures (Sakurai et al., 2008). Despite of these advances regarding the four OsPIPs (1;2, 2;1, 2;2, and 2;4) and the predominant role of OsPIP2;2 in H_2_O transport and drought tolerance, little has been known about substrate selectivity of additional seven homologs and specificities in mediating substrate transportations. The specificity of a PIP in transporting a substrate is determined by phosphorylation of the PIP at a specific serin resides in response to the substrate gradient between plant PMs (Törnroth‐Horsefield et al., 2006). Identifying the site of OsPIP2;2 phosphorylation induced by the physiological stress could explain why OsPIP2;2 is so distinct from its homologs in the physiological function.

By genetic, molecular, and physiological analyses, we elucidated that the substrate‐transporting role of OsPIP2;2 closely associates with drought tolerance in rice protoplasts and seedlings. The role of OsPIP1;2 in drought tolerance is attributable to improved maintenance of the membrane integrity and the activation of the drought tolerance pathway. Evidence has been provided as the negative effect of OsPIP2;2 on electrolyte leakage and the positive effect of OsPIP2;2 on the expression of response genes characteristic of the pathway. However, evidence obtained to date is insufficient to demonstrate molecular mechanisms underlying the functional relationship of *OsPIP2;2* between H_2_O‐transporting regulation and drought‐tolerant responses. Indeed, the physiological connection between substrate‐transporting role of PIPs and their function in drought tolerance has not been established (Oladosu et al., 2019; Shekoofa & Sinclair, 2018; Singh et al., 2020; Vinnakota et al., 2016; Vishwakarma et al., 2019). A PIP channel for water transportation mediates H_2_O trafficking in and out of plant cells, instead of one direction only inwards to the cellular interior. The assumed dynamics of oppositely directional H_2_O trafficking is accompanied by electrolyte leakage and membrane damage. Thus, the involvement of a PIP in drought tolerance is not likely to be a direct consequence from its role in H_2_O transportation. Presumably, water outflux from plant cells under an osmotic stress and enhancing role of a PIP, such as OsPIP2;2, induces physiological responses, including increases in proline and PA concentrations (Dong et al., 2004; Xu et al., 2019; Zhu et al., 2020), which turn to enhance drought resistance.

**FIGURE 11:**
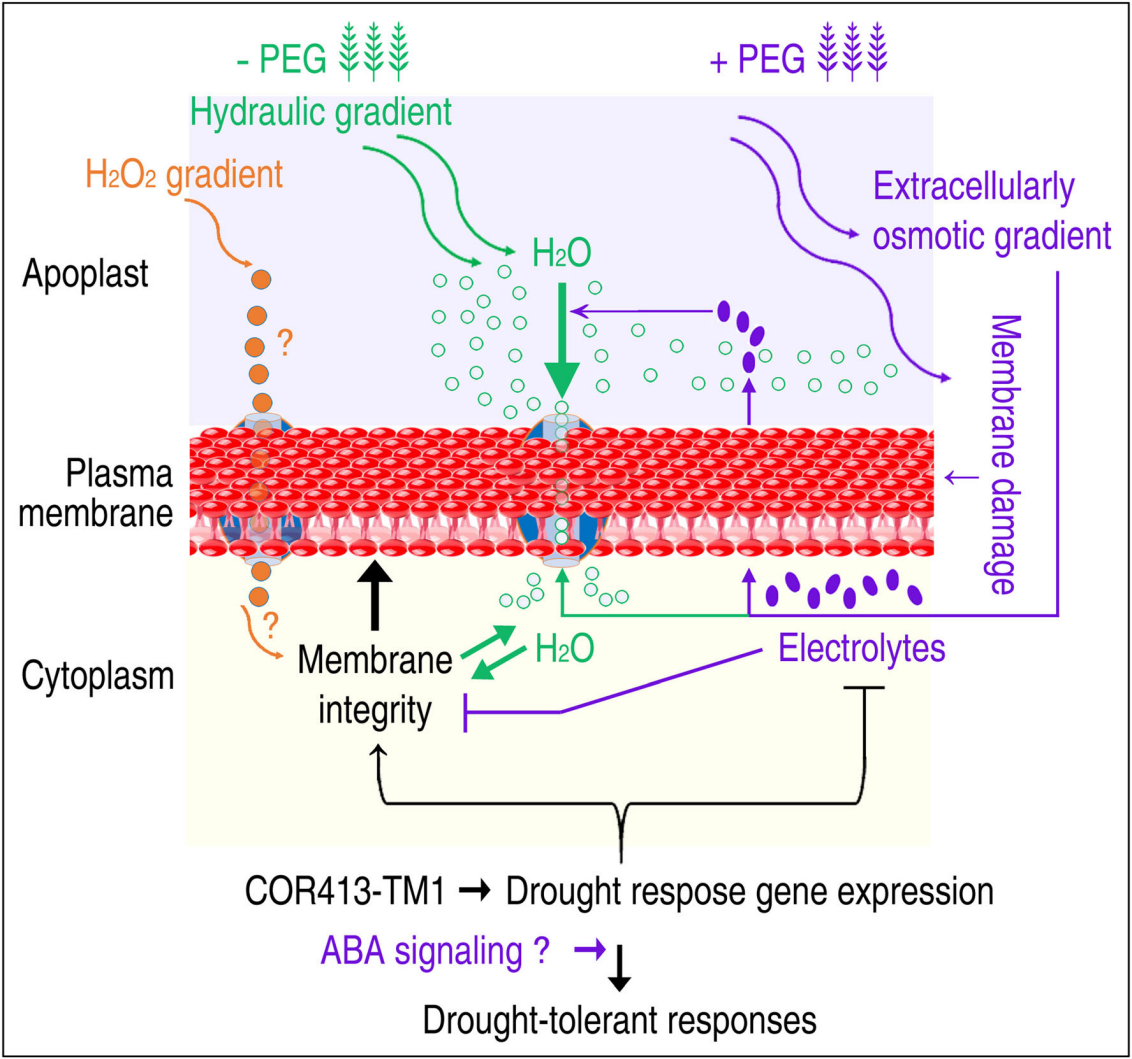
Model of OsPIP2;2 functions in H_2_O transport and drought tolerance. When plants are growing regularly without drought stress, OsPIP2;2 functions to facilitate H_2_O transport in and out of the plant cells in response to a hydraulic gradient generated by natural metabolism in the apoplastic or cytoplasmic space. This function is assumed to play a role in maintenance of water relations. When rice plants incur osmotic stress from environment, such as the physiological drought stress caused by externally applied PEG, OsPIP2;2 turns to function to support drought tolerance possibly by physical and physiological regulatory mechanisms. In the assumed physical mechanism, the drought stress is inevitable to injure the cell membranes, causing electrolyte leakage for example, while the presence of OsPIP2;2 serves as an encountering force to help preserve the membrane integrity. The physiological mechanism, including increases in proline and polyamine concentrations, is used by OsPIP2;2 to maintain the cellular water homeostasis. Water homeostasis may also come from the role of OsPIP2;2 in modulating H_2_O transportation shuttles in and out of the cells, instead of a single direction, depending on hydraulic gradient changes by electrolyte leakage. Both physical and physiological mechanisms could be integrated to increase drought tolerance intensity. Abscisic acid (ABA) signaling may partake in the regulation of OsPIP2;2‐mediated drought tolerance, which involves the ABA‐responsive transcription factor COR413‐TM1. The OsPIP2;2‐dependent tolerance pathway is also likely to have a crosstalk with signaling by H_2_O_2_ if it is induced by a particular stimulus (plant infection by a plant pathogen, for example) to accumulate in plant apoplastic spaces and to be transported by OsPIP2;2 to enter the cytoplasm

Based on these analyses, we propose a model that hypothesizes how OsPIP2;2 execute its functions toward H_2_O transport and drought tolerance (Figure 11). In the model, OsPIP2;2 supports drought tolerance by physical and physiological regulatory mechanisms, which modulate the membrane integrity by controlling the molecular (H_2_O and electrolyte) trafficking and by regulating the intracellular responses against drought stress, respectively (Figure 11). In the tested drought‐tolerant constituents, OsABF1 is a basic leucine zipper transcription factor that functions in plant responses to biotic stresses through the abscisic acid (ABA) signaling pathway. Presumably, the OsPIP2;2‐dependent drought tolerance is also subject to ABA signaling (Figure 11), which has been demonstrated to regulate plant tolerance to PEG‐induced physiological stress (Dong et al., 2005). Furthermore, characterizing the possible role of OsPIP2;2 in H_2_O_2_ transport will better explain the molecular basis of the membrane integrity. We recently proposed that H_2_O‐transporting AQPs are also potential channels for H_2_O_2_ trafficking (Wang, Schoebel, et al., 2020). In Arabidopsis, AtPIP1;4 plays triple roles in CO_2_, H_2_O (Li et al., 2015), and H_2_O_2_ transport across the PMs. H_2_O_2_ is generated in the apoplast upon induction by pathogen infection, transported by AtPIP1;4 into the cytoplasm, and therefore participates in immunity signal transduction (Tian et al., 2016). If OsPIP2;2 has a role in H_2_O_2_ transport, it may contribute to the membrane integrity by facilitating the stress‐induced apoplastic H_2_O_2_ into the cytoplasm and therefore reducing the oxidative pressure toward the cell membranes (Figure 11). Verification of these hypotheses will be the subject for further studies.

In conclusion, the physiological, molecular, and cytological performances of the WT, *OsPIP2;2*‐silencing, and *OsPIP2;2*‐overexpressing plants and protoplasts in response to the physiological drought stress demonstrate the critical role of OsPIP2;2 in H_2_O transport and drought tolerance. This finding should stimulate further studies to characterize the functional relationship of *OsPIP2;2* in H_2_O transport and drought‐tolerant responses. It is necessary to study the functional relationship between OsPIP2;1‐mediated drought tolerance and ABA signaling. It is also necessary to study if OsPIP2;10 has a role in H_2_O_2_ transport in relevance to plant defenses against biotic and abiotic stresses.

## AUTHOR CONTRIBUTIONS

J.B., X.W., L.Z., and H.D. designed the experiments. J.B., X.W., X.Y., X.C., Y.H., Z.W., and Y.M. performed the experiments and analyzed the data. X.W., L.Z., and H.D. wrote the article. L.Z. and H.D. conceived the study. All authors approved the final manuscript.

## Supporting information

**FIGURE S1** Alignments of 11 *OsPIP* coding sequences.
**FIGURE S2** Northern blotting of the OsPIP RNAs isolated from leaves of PEG_6000_‐stressed rice plants.

## References

[pld3338-bib-0001] Afzal, Z., Howton, T. C., Sun, Y., & Mukhtar, M. S. (2016). The roles of aquaporins in plant stress responses. Journal of Developmental Biology, 4, 9. 10.3390/jdb4010009 PMC5831814 29615577

[pld3338-bib-0002] Agre, P. (2004). Aquaporin water channels (Nobel Lecture). Bioscience Reports, 24, 127–163. 10.1002/anie.200460804 16209125

[pld3338-bib-0003] Agre, P., Preston, G. M., Smith, B. L., Jung, J. S., Raina, S., Moon, C., Nielsen, S., et al. (1993). Aquaporin CHIP: The archetypal molecular water channel. American Journal of Physiology, 265, F463–F476. 10.1152/ajprenal.1993.265.4.F463 7694481

[pld3338-bib-0006] Arisha, M. H., Ahmad, M. Q., Tang, W., Liu, Y., Yan, H., Kou, M., … Zhang, Y. (2020). RNA‐sequencing analysis revealed genes associated drought stress responses of different durations in hexaploid sweet potato. Scientific Reports, 10(1), 12573. 10.1038/s41598-020-69232-3 32724138 PMC7387466

[pld3338-bib-0007] Ayadi, M., Brini, F., & Masmoudi, K. (2019). Overexpression of a wheat aquaporin gene, *Td*PIP2;1, enhances salt and drought tolerance in transgenic durum wheat cv. Maali. International Journal of Molecular Science, 20(10), 2389. 10.3390/ijms20102389 PMC6566926 31091755

[pld3338-bib-0008] Barzana, G., Rios, J. J., Lopez‐Zaplana, A., Nicolas‐Espinosa, J., Yepes‐Molina, L., Garcia‐Ibañez, P., & Carvajal, M. (2020). Interrelations of nutrient and water transporters in plants under abiotic stress. Physiologia Plantarum, 10, 595–619. 10.1111/ppl.13206 32909634

[pld3338-bib-0009] Bezerra‐Neto, J. P., de Araújo, F. C., Ferreira‐Neto, J. R. C., da Silva, M. D., Pandolfi, V., Aburjaile, F. F., et al. (2019). Plant aquaporins: Diversity, evolution and biotechnological applications. Current Protein Peptide Science, 20, 368–395. 10.2174/1389203720666181102095910 30387391

[pld3338-bib-0010] Bian, H., Zhang, L., Chen, L., Wang, W., Ji, H., & Dong, H. (2019). Real‐time monitoring of translocation of selected type‐III effectors from *Xanthomonas oryzae* pv. *oryzae* into rice cells. Journal of Biosciences, 44, 82. 10.1007/s12038-019-9916-0 31502560

[pld3338-bib-0011] Bollag, W. B., Aitkens, L., White, J., & Hyndman, K. A. (2020). Aquaporin‐3 in the epidermis: More than skin deep. American Journal of Physiology Cell Physiology, 318, C1144–C1153. 10.1152/ajpcell.00075.2020 32267715 PMC7311736

[pld3338-bib-0012] Brown, D. (2017). The discovery of water channels (aquaporins). Annals of Nutrition and Metabolism, 70 Suppl 1, 37–42. 10.1159/000463061 28614812

[pld3338-bib-0013] Chen, H., Wang, G., Lu, X., Jiang, M., & Mendelssohn, I. A. (2015). Balancing the needs of China's wetland conservation and Rice production. Environmental Science Technology, 49(11), 6385–6393. 10.1021/es505988z 25955310

[pld3338-bib-0014] Ding, L., Gao, C., Li, Y., Li, Y., Zhu, Y., Xu, G., … Kai, L. (2015). The enhanced drought tolerance of rice plants under ammonium is related to aquaporin (AQP). Plant Science, 234, 14–21. 10.1016/j.plantsci.2015.01.016 25804805

[pld3338-bib-0015] Ding, L., Uehlein, N., Kaldenhoff, R., Guo, S., Zhu, Y., & Kai, L. (2019). Aquaporin *PIP2;1* affects water transport and root growth in rice (*Oryza sativa* L.). Plant Physiology and Biochemistry, 139, 152–160. 10.1016/j.plaphy.2019.03.017 30889480

[pld3338-bib-0088] Dong, H., Peng, J., Bao, Z., Meng, X., Meng, J. M., Chen, G., Beer, S. V., & Dong, H. (2004). Downstream divergence of the ethylene signaling pathway for harpin‐stimulated Arabidopsis growth and insect defense. Plant Physiology, 136(3), 3628–3638. 10.1104/pp.104.048900 15516507 PMC527161

[pld3338-bib-0016] Dong, H. P., Yu, H., Bao, Z., Guo, X., Peng, J., Yao, Z., … Qu, S. (2005). The ABI_2_‐dependent abscisic acid signalling controls HrpN‐induced drought tolerance in *Arabidopsis* . Planta, 221, 313–327. 10.1007/s00425-004-1444-x 15599761

[pld3338-bib-0017] Dubois, M., & Inzé, D. (2020). Plant growth under suboptimal water conditions: Early responses and methods to study them. Journal of Experimental Botany, 71, 1706–1722. 10.1093/jxb/eraa037 31967643

[pld3338-bib-0018] Fox, A. R., Maistriaux, L. C., & Chaumont, F. (2017). Toward understanding of the high number of plant aquaporin isoforms and multiple regulation mechanisms. Plant Science, 264, 179–187. 10.1016/j.plantsci.2017.07.021 28969798

[pld3338-bib-0086] Gomes, D., Agasse, A., Thiébaud, P., Delrot, S., Gerós, H., & Chaumont, F. (2009). Aquaporins are multifunctional water and solute transporters highly divergent in living organisms. Biochim Biophys Acta, 1788(6), 1213–1228. 19327343 10.1016/j.bbamem.2009.03.009

[pld3338-bib-0019] Grondin, A., Mauleon, R., Vadez, V., & Henry, A. (2016). Root aquaporins contribute to whole plant water fluxes under drought stress in rice (*Oryza sativa* L.). Plant Cell and Environment, 39(2), 347–365. 10.1111/pce.12616 26226878

[pld3338-bib-0020] Ha, L. (2017). Diabetes insipidus. Advances in Experimental Medicine and Biology, 969, 213–225. 10.1007/978-94-024-1057-0_14 28258576

[pld3338-bib-0087] Hachez, C., Veselov, D., Ye, Q., Reinhardt, H., Knipfer, T., Fricke, W., & Chaumont, F. (2011). Short‐term control of maize cell and root water permeability through plasma membrane aquaporin isoforms. Plant Cell Environ, 35(1), 185–198. 21950760 10.1111/j.1365-3040.2011.02429.x

[pld3338-bib-0021] Hajihashemi, S., & Geuns, J. M. (2016). Gene transcription and steviol glycoside accumulation in *Stevia rebaudiana* under polyethylene glycol‐induced drought stress in greenhouse cultivation. FEBS Open Bio, 6(9), 937–944. 10.1002/2211-5463.12099 PMC5011492 27642557

[pld3338-bib-0022] Hansen, T., Chan, A., Schröter, T., Schwerter, D., Girzalsky, W., & Erdmann, R. (2017). Isolation of native soluble and membrane‐bound protein complexes from yeast saccharomyces cerevisiae. Methods in Molecular Biology, 1595, 37–44. 10.1007/978-1-4939-6937-1_4 28409449

[pld3338-bib-0023] Hara‐Chikuma, M., Satooka, H., Watanabe, S., Honda, T., Miyachi, Y., Watanabe, T., & Verkman, A. S. (2015). Aquaporin‐3‐mediated hydrogen peroxide transport is required for NF‐κB signalling in keratinocytes and development of psoriasis. Nature Communications, 6, 7454. 10.1038/ncomms8454 PMC5628617 26100668

[pld3338-bib-0024] He, J., & Yang, B. (2019). Aquaporins in renal diseases. International Journal of Molecular Science, 20, 366. 10.3390/ijms20020366 PMC6359174 30654539

[pld3338-bib-0025] Henry, A., Cal, A. J., Batoto, T. C., Torres, R. O., & Serraj, R. (2012). Root attributes affecting water uptake of rice (*Oryza sativa*) under drought. Journal of Experimental Botany, 63(13), 4751–4763. 10.1093/jxb/ers150 22791828 PMC3427995

[pld3338-bib-0026] Hoai, P. T. T., Tyerman, S. D., Schnell, N., Tucker, M., McGaughey, S. A., Qiu, J., … Groszmann, M. (2020). Deciphering aquaporin regulation and roles in seed biology. Journal of Experimental Botany, 71, 1763–1773. 10.1093/jxb/erz555 32109278

[pld3338-bib-0028] Huang, B. L., Li, X., Liu, P., Ma, L., Wu, W., Zhang, X., … Li, Z. (2019). Transcriptomic analysis of *Eruca vesicaria* subs. *sativa* lines with contrasting tolerance to polyethylene glycol‐simulated drought stress. BMC Plant Biology, 19, 419. 10.1186/s12870-019-1997-2 31604421 PMC6787972

[pld3338-bib-0030] Ji, H., & Dong, H. (2015). Biological significance and topological basis of aquaporin‐partnering protein‐protein interactions. Plant Signaling Behavior, 10, e1011947. 10.1080/15592324.2015.1011947 26786009 PMC4854338

[pld3338-bib-0031] Jørgensen, M. E., Nour‐Eldin, H. H., & Halkier, B. A. (2016). A western blot protocol for detection of proteins heterologously expressed in *Xenopus laevis* oocytes. Methods in Molecular Biology, 1405, 99–107. 10.1007/978-1-4939-3393-8_10 26843169

[pld3338-bib-0032] Kant, R., & Dasgupta, I. (2017). Phenotyping of VIGS‐mediated gene silencing in rice using a vector derived from a DNA virus. Plant Cell Reports, 36, 1159–1170. 10.1007/s00299-017-2156-6 28540496

[pld3338-bib-0034] Kirscht, A., Survery, S., Kjellbom, P., & Johanson, U. (2016). Increased permeability of the aquaporin SoPIP2;1 by mercury and mutations in loop a. Frontiers in Plant Science, 7, 1249. 10.3389/fpls.2016.01249 27625657 PMC5004352

[pld3338-bib-0035] Kromdijk, J., Głowacka, K., & Long, S. P. (2020). Photosynthetic efficiency and mesophyll conductance are unaffected in *Arabidopsis thaliana* aquaporin knock‐out lines. Journal of Experimental Botany, 71, 318–329. 10.1093/jxb/erz442 31731291

[pld3338-bib-0036] Kukulski, W., Schenk, A. D., Johanson, U., Braun, T., de Groot, B. L., Fotiadis, D., Kjellbom, P., & Engel, A. (2005). The 5A structure of heterologously expressed plant aquaporin SoPIP2;1. Journal of Molecular Biology, 350, 611–616. 10.1016/j.jmb.2005.05.001 15964017

[pld3338-bib-0037] Laloux, T., Junqueira, B., Maistriaux, L., Ahmed, J., Jurkiewicz, A., & Chaumont, F. (2018). Plant and mammal aquaporins: Same but different. International Journal of Molecular Science, 19, 521. 10.3390/ijms19020521 PMC5855743 29419811

[pld3338-bib-0038] de Laurentis, C., Cristaldi, P., Arighi, A., Cavandoli, C., Trezza, A., Sganzerla, E. P., et al. (2020). Role of aquaporins in hydrocephalus: What do we know and where do we stand? A Systematic Review. Journal of Neurology. 10.1007/s00415-020-10122-z 32747978

[pld3338-bib-0040] Lee, K. S., Choi, W. Y., Ko, J. C., Kim, T. S., & Gregorio, G. B. (2003). Salinity tolerance of japonica and indica rice (*Oryza sativa* L.) at the seedling stage. Planta, 216(6), 1043–1046. 10.1007/s00425-002-0958-3 12687373

[pld3338-bib-0041] Li, C., & Wang, W. (2017). Molecular biology of aquaporins. Advances in Experimental Medicine and Biology, 969, 1–34. 10.1007/978-94-024-1057-0_1 28258563

[pld3338-bib-0042] Li, L., Wang, H., Gago, J., Cui, H., Qian, Z., Kodama, N., Dong, H., et al. (2015). Harpin Hpa1 interacts with aquaporin PIP1;4 to promote the substrate transport and photosynthesis in *Arabidopsis* . Scientific Reports, 10, 1038. 10.1038/srep17207 PMC4660436 26607179

[pld3338-bib-0043] Li, P., Zhang, L., Mo, X., Ji, H., Bian, H., Hu, Y., … Ma, J. (2019). Aquaporin PIP1;3 of rice and harpin Hpa1 of bacterial blight pathogen cooperate in a type III effector translocation. Journal of Experimental Botany, 70, 3057–3073. 10.1093/jxb/erz130 30921464 PMC6598099

[pld3338-bib-0045] Liu, R., Chen, L., Jia, Z., Lü, B., Shi, H., Shao, W., & Dong, H. (2011). Transcription factor AtMYB44 regulates induced expression of the ETHYLENE INSENSITIVE2 gene in Arabidopsis responding to a harpin protein. Molecular Plant Microbe Interactions, 24, 377–389. 10.1094/MPMI-07-10-0170 21117868

[pld3338-bib-0046] Livak, K. J., & Schmittgen, T. D. (2011). Analysis of relative gene expression data using real‐time quantitative PCR and the 2^−ΔΔ*C* ^ _T_ method. Methods, 25, 402–408. 10.1006/meth.2001.1262 11846609

[pld3338-bib-0047] Mañosa, S., Mateo, R., & Guitart, R. (2001). A review of the effects of agricultural and industrial contamination on the Ebro delta biota and wildlife. Environmental Monitoring and Assessment, 71(2), 187–205. 10.1023/a:1017545932219 11686200

[pld3338-bib-0048] Maurel, C., Boursiac, Y., Luu, D. T., Santoni, V., Shahzad, Z., & Verdoucq, L. (2015a). Aquaporins in plants. Physiological Reviews, 95, 1321–1358. 10.1152/physrev.00008.2015 26336033

[pld3338-bib-0049] Maurel, C., Boursiac, Y., Luu, D. T., Santoni, V., Shahzad, Z., & Verdoucq, L. (2015b). Aquaporins in plants. Physiology, 95, 1321–1358. 10.1152/physrev.00008.2015 26336033

[pld3338-bib-0090] Maurel, C., Verdoucq, L., Luu, D. T., & Santoni, V. (2008). Plant aquaporins: membrane channels with multiple integrated functions. Annu Rev Plant Biol, 59, 595–624. 10.1146/annurev.arplant.59.032607.092734 18444909

[pld3338-bib-0050] Nada, R. M., & Abogadallah, G. M. (2020). Contrasting root traits and native regulation of aquaporin differentially determine the outcome of overexpressing a single aquaporin (OsPIP2;4) in two rice cultivars. Protoplasma, 257(2), 583–595. 10.1007/s00709-019-01468-x 31840193

[pld3338-bib-0052] Oladosu, Y., Rafii, M. Y., Samuel, C., Fatai, A., Magaji, U., Kareem, I., … Muhammad, I.’. (2019). Drought resistance in rice from conventional to molecular breeding: A review. International Journal of Molecular Science, 20, 3519. 10.3390/ijms20143519 PMC6678081 31323764

[pld3338-bib-0091] Péret, B., Li, G., Zhao, J., Band, L. R., Voß, U., Postaire, O., Luu, D. T., Da Ines, O., Casimiro, I., Lucas, M., Wells, D. M., Lazzerini, L., Nacry, P., King, J. R., Jensen, O. E., Schäffner, A. R., Maurel, C., & Bennett, M. J. (2012). Auxin regulates aquaporin function to facilitate lateral root emergence. Nature Cell Biology, 14, 991–998. 10.1038/ncb2573 22983115

[pld3338-bib-0053] Plett, D. C., Ranathunge, K., Melino, V. J., Kuya, N., Uga, Y., & Kronzucker, H. J. (2020). The intersection of nitrogen nutrition and water use in plants: New paths toward improved crop productivity. Journal of Experimental Botany, 71, 4452–4468. 10.1093/jxb/eraa049 32026944 PMC7382376

[pld3338-bib-0054] Preston, G. M., Carroll, T. P., Guggino, W. B., & Agre, P. (1992). Appearance of water channels in *Xenopus* oocytes expressing red cell CHIP28 protein. Science, 256, 385–387. 10.1126/science.256.5055.385 1373524

[pld3338-bib-0055] Purkayastha, A., Mathur, S., Verma, V., Sharma, S., & Dasgupta, I. (2010). Virus‐induced gene silencing in rice using a vector derived from a DNA virus. Planta, 232, 1531–1540. 10.1007/s00425-010-1273-z 20872012

[pld3338-bib-0056] Ren, Y. R., Yang, Y. Y., Zhang, R., You, C. X., Zhao, Q., & Hao, Y. J. (2019). MdGRF11, an apple 14‐3‐3 protein, acts as a positive regulator of drought and salt tolerance. Plant Science, 288, 110219. 10.1016/j.plantsci.2019.110219 31521216

[pld3338-bib-0057] Rhee, J., Horie, T., Sasano, S., Nakahara, Y., & Katsuhara, M. (2017). Identification of an H_2_O_2_ permeable PIP aquaporin in barley and a serine residue promoting H_2_O_2_ transport. Physiologia Plantarum, 159, 120–128. 10.1111/ppl.12508 27595571

[pld3338-bib-0058] Sadura, I., Libik‐Konieczny, M., Jurczyk, B., Gruszka, D., & Janeczko, A. (2020). Plasma membrane ATPase and the aquaporin HvPIP1 in barley brassinosteroid mutants acclimated to high and low temperature. Journal of Plant Physiology, 244, 153090. 10.1016/j.jplph.2019.153090 31841952

[pld3338-bib-0089] Sakurai, J., Ahamed, A., Murai, M., Maeshima, M., & Uemura, M. (2008). Tissue and cell‐specific localization of rice aquaporins and their water transport activities. Plant and Cell Physiology, 49, 30–39. 10.1093/pcp/pcm162 18037610

[pld3338-bib-0059] Sakurai, J., Ishikawa, F., Yamaguchi, T., Uemura, M., & Maeshima, M. (2005). Identification of 33 rice aquaporin genes and analysis of their expression and function. Plant Cell Physiology, 46, 1568–1577. 10.1093/pcp/pci172 16033806

[pld3338-bib-0060] Shekoofa, A., & Sinclair, T. R. (2018). Aquaporin activity to improve crop drought tolerance. Cell, 7, 123. 10.3390/cells7090123 PMC6162707 30158445

[pld3338-bib-0061] Shen, J., Fu, J., Ma, J., Wang, X., Gao, C., Zhuang, C., … Wan, J. (2014). Isolation, culture, and transient transformation of plant protoplasts. Current Protocols in Cell Biology, 63, 2.8.1–2.8.17. 10.1002/0471143030.cb0208s63 24894837

[pld3338-bib-0062] Shi, L. W. (2012). SPSS19.0 Statistical Analysis From Accidence to Conversance (in Chinese) (pp. 109–143). Beijing: Tsinghua University Press.

[pld3338-bib-0063] Singh, R. K., Deshmukh, R., Muthamilarasan, M., Rani, R., & Prasad, M. (2020). Versatile roles of aquaporin in physiological processes and stress tolerance in plants. Plant Physiology and Biochemistry, 149, 178–189. 10.1016/j.plaphy.2020.02.009 32078896

[pld3338-bib-0064] Sriskantharajah, K., Osumi, S., Chuamnakthong, S., Nampei, M., Amas, J. C., Gregorio, G. B., & Ueda, A. (2020). Acquired salinity tolerance in rice: Plant growth dataset. Data in Brief, 31, 106023. 10.1016/j.dib.2020.106023 32728604 PMC7381506

[pld3338-bib-0065] Tian, S., Wang, X., Li, P., Wang, H., Ji, H., Xie, J., Dong, H., et al. (2016). Plant aquaporin AtPIP1;4 links apoplastic H_2_O_2_ induction to disease immunity pathways. Plant Physiology, 171, 1635–1650. 10.1104/pp.15.01237 26945050 PMC4936539

[pld3338-bib-0066] Tiwari, P., Srivastava, D., Chauhan, A. S., Indoliya, Y., Singh, P. K., Tiwari, S., … Chauhan, P. S. (2020). Root system architecture, physiological analysis and dynamic transcriptomics unravel the drought‐responsive traits in rice genotypes. Ecotoxicology and Environmental Safety, 207, 111252. 10.1016/j.ecoenv.2020.111252 32916530

[pld3338-bib-0067] Törnroth‐Horsefield, S., Wang, Y., Hedfalk, K., Johanson, U., Karlsson, M., Tajkhorshid, E., Neutze, R., & Kjellbom, P. (2006). Structural mechanism of plant aquaporin gating. Nature, 439, 688–694. 10.1038/nature04316 16340961

[pld3338-bib-0068] Vinnakota, R., Ramakrishnan, A. M., Samdani, A., Venugopal, M. A., Ram, B. S., Krishnan, S. N., … Murugesan, D. (2016). A comparison of aquaporin function in mediating stomatal aperture gating among drought‐tolerant and sensitive varieties of rice (*Oryza sativa* L.). Protoplasma, 253, 1593–1597. 10.1007/s00709-015-0916-0 26631017

[pld3338-bib-0069] Vishwakarma, K., Mishra, M., Patil, G., Mulkey, S., Ramawat, N., Singh, V. P., et al. (2019). Avenues of the membrane transport system in adaptation of plants to abiotic stresses. Critical Reviews in Biotechnology, 39, 861–883. 10.1080/07388551.2019.1616669 31362527

[pld3338-bib-0070] Wang, H., Schoebel, S., Schmitz, F., Dong, H., & Hedfalk, K. (2020). Characterization of aquaporin‐driven hydrogen peroxide transport. Biochimica Et Biophysica Acta‐ Biomembranes, 1862, 183065. 10.1016/j.bbamem.2019.183065 31521632

[pld3338-bib-0071] Wang, H., Zhang, L., Tao, Y., Wang, Z., Shen, D., & Dong, H. (2020). Transmembrane helices 2 and 3 determine the localization of plasma membrane intrinsic proteins in eukaryotic cells. Frontiers in Plant Science, 10, 1671. 10.3389/fpls.2019.01671 31998350 PMC6966961

[pld3338-bib-0072] Wang, R., Wang, M., Chen, K., Wang, S., Mur, L. A. J., & Guo, S. (2018). Exploring the roles of aquaporins in plant‐microbe interactions. Cell, 12, 267. 10.3390/cells7120267 PMC6316839 30545006

[pld3338-bib-0073] Wang, X., Zhang, L., Ji, H., Mo, X., Li, P., Wang, J., Dong, H., et al. (2018). Hpa1 is a type III translocator in *Xanthomonas oryzae* pv. *oryzae* . BMC Microbiology, 18, 105. 10.1186/s12866-018-1251-3 30180793 PMC6123991

[pld3338-bib-0074] Withers, K. (2002). Shorebird use of coastal wetland and barrier island habitat in the Gulf of Mexico. Scientific World Journal, 2, 514–536. 10.1100/tsw.2002.112 12806034 PMC6009297

[pld3338-bib-0075] Xu, F., Wang, K., Yuan, W., Xu, W., Shuang, L., Kronzucker, H. J., et al. (2019). Overexpression of rice aquaporin *OsPIP1;2* improves yield by enhancing mesophyll CO_2_ conductance and phloem sucrose transport. Journal of Experimental Botany, 70, 671–681. 10.1093/jxb/ery386 30535321 PMC6322580

[pld3338-bib-0076] Yoo, S., Cho, Y. H., & Sheen, J. (2007). Arabidopsis mesophyll protoplasts, a versatile cell system for transient gene expression analysis. Nature Protocols, 2, 1565–1572. 10.1038/nprot.2007.199 17585298

[pld3338-bib-0077] Zhang, C., Li, C., Liu, J., Lv, Y., Yu, C., Li, H., Zhao, T., & Liu, B. (2017). The OsABF1 transcription factor improves drought tolerance by activating the transcription of COR413‐TM1 in rice. Journal of Experimental Botany, 68(16), 4695–4707. 10.1093/jxb/erx260 28981779 PMC5853872

[pld3338-bib-0079] Zhang, L., Chen, L., & Dong, H. (2019). Plant aquaporins in infection by and immunity against pathogens – A critical review. Frontiers in Plant Science, 10, 632. 10.3389/fpls.2019.00632 31191567 PMC6546722

[pld3338-bib-0080] Zhang, L., Hu, Y., Li, P., Wang, X., & Dong, H. (2019). Silencing of an aquaporin gene diminishes bacterial blight disease in rice. Australasian Plant Pathology, 48, 143–158. 10.1007/s13313-018-0609-1

[pld3338-bib-0083] Zhu, M. D., Zhang, M., Gao, D. J., Zhou, K., Tang, S. J., Zhou, B., & Lv, Y. M. (2020). Rice *OsHSFA3* gene improves drought tolerance by modulating polyamine biosynthesis depending on abscisic acid and ROS levels. International Journal of Molecular Science, 21, 1857. 10.3390/ijms21051857 PMC7084839 32182761

[pld3338-bib-0084] Zong, Y., Chen, Z., Innes, J. B., Chen, C., Wang, Z., & Wang, H. (2007). Fire and flood management of coastal swamp enabled first rice paddy cultivation in east China. Nature, 449(7161), 459–462. 10.1038/nature06135 17898767

