# Supplementary material for: Rice aquaporin OsPIP2;2 is a water‐transporting facilitator in relevance to drought‐tolerant responses
Source: Plant Direct. 2021 Aug 16;5(8):e338. doi: 10.1002/pld3.338 (PMC8365552; doi:10.1002/pld3.338)
Supplement: Supplementary file 1 — FIGURE S1 Alignments of 11 OsPIP coding sequences. FIGURE S2 Northern blotting of the OsPIP RNAs isolated from leaves of PEG6000‐stressed rice plants. [file PLD3-5-e338-s001.doc]

**Rice Aquaporin OsPIP2;2** **Is a Water-Transporting Facilitator in Relevance to Drought-Tolerant Responses**

Jiaqi Bai | Xuan Wang | Xiaohui Yao | Xiaochen Chen | Kai Lu | Yiqun Hu | Zuodong Wang | Yanjie Mu | Liyuan Zhang | Hansong Dong

**Supplemental Data**


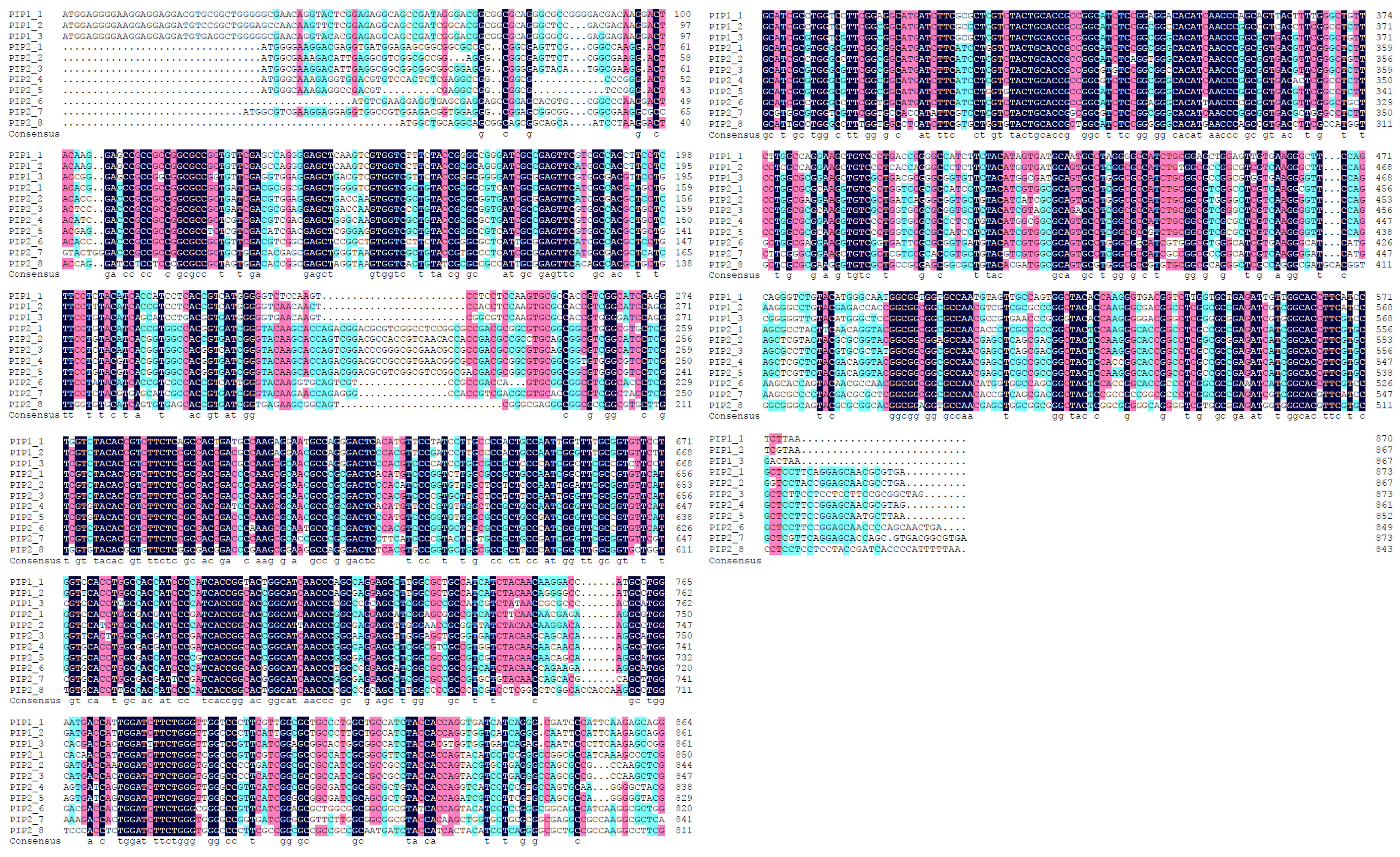


**FIGURE S1** Alignments of 11 *OsPIP* coding sequences.


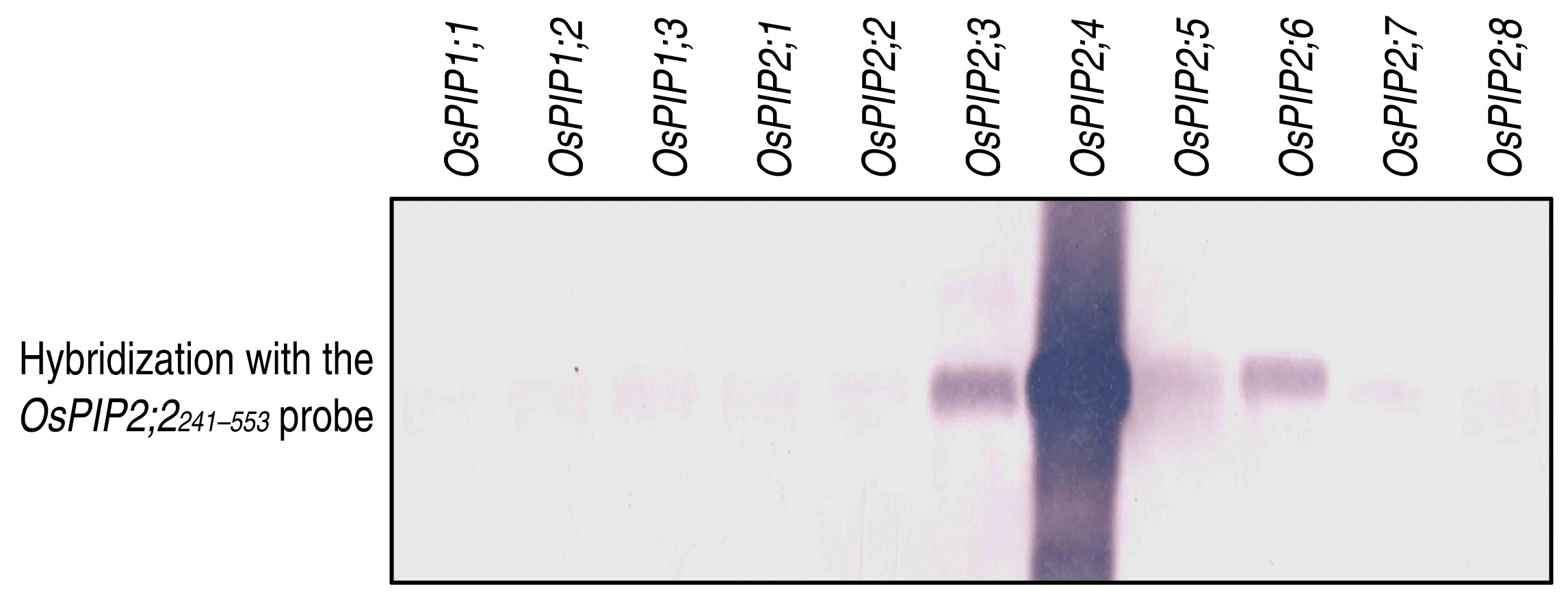


**FIGURE S2** Northern blotting of the OsPIP RNAs isolated from leaves of PEG_6000_-stressed rice plants.
